# Protein profile of *Beta vulgaris* leaf apoplastic fluid and changes induced by Fe deficiency and Fe resupply

**DOI:** 10.3389/fpls.2015.00145

**Published:** 2015-03-18

**Authors:** Laura Ceballos-Laita, Elain Gutierrez-Carbonell, Giuseppe Lattanzio, Saul Vázquez, Bruno Contreras-Moreira, Anunciación Abadía, Javier Abadía, Ana-Flor López-Millán

**Affiliations:** ^1^Plant Stress Physiology Group, Department of Plant Nutrition, Aula Dei Experimental Station, Consejo Superior de Investigaciones CientíficasZaragoza, Spain; ^2^Laboratory of Computational and Structural Biology, Aula Dei Experimental Station, Consejo Superior de Investigaciones CientíficasZaragoza, Spain; ^3^Fundación ARAIDZaragoza, Spain

**Keywords:** leaf apoplast, iron deficiency, proteome, sugar beet, two-dimensional electrophoresis

## Abstract

The fluid collected by direct leaf centrifugation has been used to study the proteome of the sugar beet apoplastic fluid as well as the changes induced by Fe deficiency and Fe resupply to Fe-deficient plants in the protein profile. Plants were grown in Fe-sufficient and Fe-deficient conditions, and Fe resupply was carried out with 45 μM Fe(III)-EDTA for 24 h. Protein extracts of leaf apoplastic fluid were analyzed by two-dimensional isoelectric focusing-SDS-PAGE electrophoresis. Gel image analysis revealed 203 consistent spots, and proteins in 81% of them (164) were identified by nLC-MS/MS using a custom made reference repository of beet protein sequences. When redundant UniProt entries were deleted, a non-redundant leaf apoplastic proteome consisting of 109 proteins was obtained. TargetP and SecretomeP algorithms predicted that 63% of them were secretory proteins. Functional classification of the non-redundant proteins indicated that stress and defense, protein metabolism, cell wall and C metabolism accounted for approximately 75% of the identified proteome. The effects of Fe-deficiency on the leaf apoplast proteome were limited, with only five spots (2.5%) changing in relative abundance, thus suggesting that protein homeostasis in the leaf apoplast fluid is well-maintained upon Fe shortage. The identification of three chitinase isoforms among proteins increasing in relative abundance with Fe-deficiency suggests that one of the few effects of Fe deficiency in the leaf apoplast proteome includes cell wall modifications. Iron resupply to Fe deficient plants changed the relative abundance of 16 spots when compared to either Fe-sufficient or Fe-deficient samples. Proteins identified in these spots can be broadly classified as those responding to Fe-resupply, which included defense and cell wall related proteins, and non-responsive, which are mainly protein metabolism related proteins and whose changes in relative abundance followed the same trend as with Fe-deficiency.

## Introduction

Iron is the fourth most abundant element in the earth's crust and it is an essential micronutrient for all living organisms including plants. However, its low availability in neutral or alkaline soils, which account for approximately 30% of the world's arable soils, causes Fe deficiency (Abadía et al., 2004). Iron deficiency is the most common nutritional disorder in many plants and typical symptoms include chlorosis of young leaves (leaf yellowing) and reduced crop yield and quality, which result in increased orchard management costs (Álvarez-Fernández et al., 2006; Rombolà and Tagliavini, 2006; Abadía et al., 2011).

Plants have developed two main mechanisms to allow Fe uptake from the soil: a strategy based on Fe (III) chelation (Strategy II) used by graminaceous plants, and a strategy used by non-graminaceous plants based on Fe(III) reduction (Strategy I) (Römheld and Marschner, 1986; Curie and Briat, 2003; Abadía et al., 2011). When challenged with Fe shortage, Strategy I plants such as *Beta vulgaris* increase the activity of several enzymes at the root plasma membrane level. These changes are aimed at increasing Fe uptake and include increases in a Fe(III) reductase (FRO, Ferric Reductase Oxidase; Robinson et al., 1999), an Fe transporter (IRT1, Iron Regulated Transporter) which introduces Fe(II) into the root cell (Eide et al., 1996; Fox and Guerinot, 1998) and an H^+^-ATPase that lowers the pH of the rhizosphere increasing soil Fe solubility (Santi et al., 2005; Santi and Schmidt, 2008, 2009). Also, several changes occur at the metabolic level in order to support the increased demand of energy and reducing power of Fe-deficient Strategy I roots (Zocchi, 2006). These changes include increased activity of the glycolytic pathway and TCA cycle, shifts in the redox state of the cytoplasm and in the mitochondrial electron transport chain (Schmidt, 1999; López-Millán et al., 2000b; Zocchi, 2006; Vigani, 2012).

While it is well-known that Fe is transported to the shoot *via* xylem, complexed by citrate (López-Millán et al., 2000a; Rellán-Álvarez et al., 2010), the mechanisms for Fe loading and unloading from the vasculature system are not yet fully understood. These processes could take place *via* parenchyma cells or by passive diffusion to the apoplastic space driven by transpiration (Abadía et al., 2011). Also, Fe uptake by mesophyll cells is not as well-studied as in roots. An Fe-reductase activity has been detected in leaf cells and protoplasts (Nikolic and Römheld, 1999; González-Vallejo et al., 2000; Jeong and Connolly, 2009) and AtFRO6 has been located in leaf PM-membranes (Mukherjee et al., 2006; Jeong et al., 2008). However, *fro6* mutant plants do not display any Fe-deficiency symptoms (Jeong and Connolly, 2009) therefore suggesting the existence of other reducing mechanisms. Factors such as differences in apoplastic pH and carboxylate concentrations as a result of Fe deficiency may also regulate leaf Fe reductase activity. On the other hand, light has also been proposed to directly photoreduce Fe (III)-citrate complexes in the leaf apoplast (Nikolic and Römheld, 2007).

The apoplast is a free diffusional space outside the plasma membrane that occupies less of 5% of the plant tissue volume in aerial organs (Steudle et al., 1980; Parkhurst, 1982) and the root cortex (Vakhmistrov, 1967). Among other important functions, such as transport and storage of minerals (Starrach and Mayer, 1989; Wolf et al., 1990; Zhang et al., 1991) or signal transmission (Hartung et al., 1992), the apoplast plays a major role in plant defense (Pechanova et al., 2010). Given that the composition of the apoplastic fluid results from the balance between xylem and phloem transport and mesophyll cell uptake processes, small changes in these fluxes could produce large changes in the solute concentrations in the apoplast. Changes in the apoplastic composition have been described in biotic and abiotic stresses such as Fe deficiency, air pollutants, heavy metal toxicity, drought, salinity, and extreme temperature (Griffith et al., 1992; Brune et al., 1994; Covarrubias et al., 1995; Dietz, 1997; López-Millán et al., 2000a; Fecht-Christoffers et al., 2003). For instance, Fe deficiency causes a slight decrease in the pH of the apoplast and has a strong impact on the carboxylate composition, with major increases in the concentrations of citrate and malate (López-Millán et al., 2000a). These Fe-deficiency induced changes in the leaf apoplast chemical environment have been suggested to play a role in Fe homeostasis (López-Millán et al., 2000a).

Apoplastic fluid isolation is always carried out using some degree of force (e.g., vacuum perfusion, leaf centrifugation, or pressure using a Schölander bomb), therefore leading to the presence of some cytosolic components in the samples (Lohaus et al., 2001). This contamination may be originated by the rupture of a certain fraction of the leaf mesophyll cells, or, alternatively, by the rupture of the plasmodesmata that communicate neighboring cells. The degree of contamination is usually assessed using cellular marker enzymes such as cytosolic malate deshidrogenase (c-mdh) or other cytoplasmic or internal organelle components, with values ≤3% considered acceptable (Dannel et al., 1995; Lohaus et al., 2001).

Proteomic approaches are useful to understand the general effect that a given stress exerts on metabolic processes (Jorrín-Novo et al., 2009). These approaches have been previously used to study the effects of Fe deficiency in several plant tissues, including thylakoids and roots (López-Millán et al., 2013). Most of the leaf apoplastic proteomic studies so far have been devoted to the study of the protein profile or the effect of biotic stresses. However, knowledge about the effects of nutritional stresses such as Fe deficiency in the apoplastic fluid protein profile is still very limited. Therefore, the aim of this study was first to obtain an overview of the leaf apoplast proteome in sugar beet plants and second to characterize the changes induced by Fe deficiency and resupply in the protein profile of this compartment, with the final goal of shedding light into the major processes taking place in the apoplast and the effect of Fe deficiency on them.

## Material and methods

### Plant material and growth conditions

Sugar beet (*Beta vulgaris* L. cv. Orbis) was grown in a growth chamber with a photosynthetic photon flux density (PPFD) of 350 μmol m^−2^ s^−1^ PAR, 80% relative humidity and a photoperiod of 16 h, 23°C/8 h, 18°C day/night regime. Seeds were germinated and grown in vermiculite for 2 weeks. Seedlings were grown for an additional 2 weeks period in half-strength Hoagland nutrient solution with 45 μM Fe(III)-EDTA, and then transplanted to 20 L plastic buckets (four plants per bucket) containing half-strength Hoagland nutrient solution with either 0 or 45 μM Fe(III)-EDTA. Iron-free nutrient solutions were buffered at pH 7.7 with 1 mM NaOH and 1 g L^−1^ of CaCO_3_. Young leaves from plants grown for 10 d in the presence and absence of Fe were used to collect the apoplastic fluid in all experiments. In the Fe-resupply experiment, 45 μM Fe (III)-EDTA was added to the nutrient solution of plants grown for 10 d in the absence of Fe. The apoplastic fluid of these plants was collected 24 h after Fe addition.

### Experimental design

The experiment was repeated four times with independent sets of plants. Each batch of plants consisted of four buckets per treatment with four plants per bucket. Apoplastic fluid was isolated from the four to five youngest leaves of each plant. Cytosolic contamination was assayed in each sample as described below. Samples with less than 3% of cytosolic contamination from a given treatment per batch were pooled together and considered as a biological replicate (*n* = 4).

### Collection of leaf apoplastic fluid

Leaf apoplastic fluid was obtained from whole sugar beet leaves by direct centrifugation (López-Millán et al., 2000a). Briefly, leaves were excised at the base of the petiole and each leaf was rolled and placed into a plastic syringe barrel. Leaf-filled syringes were first centrifuged at low speed (2500 g, 4°C, 15 min) to remove the xylem sap from the main vein and the fluid collected was discarded. A second centrifugation was then carried out at 4°C, 4000 g for 15 min and the fluid collected was considered as soluble apoplastic fluid. The activity of cytosolic malate dehydrogenase (c-mdh; EC 1.1.1.37) in the collected fluid was measured immediately and used as a cytosolic contamination marker. The activity of c-mdh was measured spectrophotometrically at 340 nm in a final reaction mixture containing 46.5 mM Tris (pH 9.5), 0.1 mM NADH, 0.4 mM oxaloacetate and 5 μL of apoplastic fluid and referred to activity measured in a whole leaf extract (López-Millán et al., 2000a). For the activity in whole leaf extracts, approximately 0.1 g of leaf tissue was homogenized with 2 mL of a buffer (pH 8.0) containing 100 mM HEPES, 30 mM sorbitol, 2 mM DTT, 1 mM CaCl_2_, 1% (w/v) bovine serum albumin and 1% (w/v) polyvinylpyrrolidone. The supernatant was collected and analyzed immediately after a 10 min centrifugation at 10,000 g.

### Protein extraction

Proteins in approximately 1 mL of apoplastic fluid were precipitated by adding five volumes of cold 10% TCA. Samples were incubated for at least 14 h at 4°C and then centrifuged at 10,000 g for 15 min. The pellet was washed twice with cold methanol, dried with N_2_ gas and solubilized in a sample rehydration buffer containing 8 M urea, 2% (w/v) CHAPS, 50 mM DTT, 2 mM PMSF and 0.2% (v/v) IPG buffer pH 3–10 (GE Healthcare, Uppsala, Sweden). After rehydration, samples were incubated in a Thermomixer Confort device (Eppendorf AG, Hamburg, Germany) at 29°C and 1000 rpm during 3 h, then centrifuged at 10,000 ×*g* for 10 min at RT and filtered (0.45 μm ultrafree-MC filters, Millipore, Bedford, USA). Protein concentration in the samples was quantified immediately with the Bradford method in microtiter plates using an Asys UVM 340 spectrophotometer (Biochrom Ltd., Cambridge, UK) and BSA as standard.

### 2-DE

A first dimension IEF separation was carried out on 7 cm ReadyStrip IPG Strips (BioRad, Hercules, CA, USA) with a linear pH gradient 3–10 in a Protean IEF Cell (BioRad). Strips were passively rehydrated for 16 h at 20°C in 125 μL of rehydration buffer containing 80 μg of apoplast proteins and a trace of bromophenol blue and then strips were transferred onto a strip electrophoresis tray. IEF was run at 20°C, for a total of 14,000 V h (20 min with 0–250 V linear gradient; 2 h with 250–4000 V linear gradient and 4000 V until 10,000 V h). After IEF, strips were equilibrated for 10 min in equilibration solution I [6 M urea, 0.375 M Tris-HCl, pH 8.8, 2% (w/v) SDS, 20% (v/v) glycerol, 2% (w/v) DTT] and for another 10 min in equilibration solution II [6 M urea, 0.375 M Tris-HCl pH 8.8, 2% (w/v) SDS, 20% (v/v) glycerol, 2.5% (w/v) iodoacetamide]. For the second dimension, polyacrylamide gel electrophoresis (SDS-PAGE) and equilibrated IPG strips were placed on top of vertical 12% SDS-polyacrylamide gels (8 × 10 × 0.1 cm) and sealed with melted 0.5% agarose in 50 mM Tris-HCl (pH 6.8) containing 0.1% SDS. SDS-PAGE was carried out at 20 mA per gel for approximately 1.5 h at 4°C, until the bromophenol blue reached the plate bottom, in a buffer containing 25 mM Tris Base, 192 mM glycine, and 0.1% SDS. Gels were subsequently stained with Coomassie blue G-250 (Serva, Barcelona, Spain). For each treatment, gels were made from four independent apoplast protein extracts (*n* = 4), each of them obtained by pooling the apoplastic fluid collected from 5 to 6 leaves in a given batch.

### Gel image and statistical analysis

Stained gels were scanned with an Epson Perfection 4990 Photo Scanner (Epson Ibérica, Barcelona, Spain), previously calibrated using the SilverFast 6 software (LaserSoft Imaging AG, Kiel, Germany) using an IT8 reference card. Experimental MR values were calculated by mobility comparisons with Precision Plus protein standard markers (BioRad) run in a separate lane on the SDS-gel, and pI was determined by using a 3–10 linear scale over the total dimension of the IPG strips. Spot detection, gel matching and interclass analysis were performed with PDQuest 8.0 software (BioRad). Normalized spot volumes based on total intensity of valid spots were calculated for each 2-DE gel and used for statistical calculations of protein abundance; for all spots present in the gels, pI, Mr, and normalized volumes (mean values and SD) were determined. Only spots present in all four replicates from at least one treatment were considered as consistent and used in further analysis. The spots were also manually checked, and a high level of reproducibility between normalized spot volumes was found in all four different replicates.

Univariate and multivariate statistical analyses were carried out. Protein response ratios were defined as the relative abundance in a given treatment divided by the relative abundance in the control; when ratios were lower than one the inverse was taken and the sign changed. Spots changing in relative abundance were defined using a Student *t*-test and a significance level of *p* < 0.05. Among these, only protein species with mean response ratios above 2.0 or below −2.0 were considered relevant and are discussed in this study. Principal component analysis (PCA) analysis was also carried out, using Statistical software (v. 10.0) and including only those spots showing differential accumulation as a result of the Fe-deficiency and Fe-resupply treatments.

### Protein in gel digestion

Consistent spots were excised automatically using a spot cutter EXQuest (BioRad), transferred to 500 μL Protein LoBind Eppendorf tubes, distained in 400 μL of 40% [v/v] acetonitrile (ACN) and 60% [v/v] 200 mM NH_4_HCO_3_ for 30 min and dehydrated in 100% ACN for 10 min. Gel pieces were dried at RT and then *in gel* digested with 15 μL Trypsin solution (Sequencing Grade Modified Trypsin V511, Promega, Madison, WI, US; 0.1 μg μL^−1^ in 40 mM NH_4_HCO_3_/9% ACN). After incubation for 5 h at 37°C, the reaction was stopped by adding 1 μL of 1% TFA. The peptide solution was finally analyzed using mass spectrometry (MS).

### Reference repository of beet protein sequences

Proteomes of five sequenced beet accessions (RefBeet, KDHBv, YMoBv, UMSBv and YTiBv) were downloaded from http://bvseq.molgen.mpg.de/Genome/Download, corresponding to gene models annotated as of February 2013. In addition, all *B. vulgaris* protein sequences annotated in Uniprot (www.uniprot.org) were retrieved, and added to the set, which was subsequently filtered to remove redundant sequences with software CD-HIT (http://www.ncbi.nlm.nih.gov/pubmed/23060610) with cutoff -c 1.0 and otherwise default parameters. The final non-redundant set contained 82,368 protein sequences.

### Protein identification by nano-liquid chromatography-tandem mass spectrometry (nLC-ESI-MS/MS)

Peptides present in 6 μL of sample were pre-concentrated on line onto a 300 μm i.d. × 5 mm, 5 μm particle size ZORBAX 300SB-C18 trap column (Agilent Technologies, Waldbronn, Germany), using a 100 μL min^−1^ flow rate of 3% ACN, 0.1% formic acid, in a nano-HPLC system 1200 series (Agilent Technologies). Backflow elution of peptides from the trap column was carried out, and separation was done with a 75 μm i.d. × 150 mm, 3.5 μm particle size ZORBAX 300SB-C18 column (Agilent Technologies), using a 300 nL min^−1^ nano-flow rate and a 55 min linear gradient from solution 97% A (0.1% formic acid) to 90% of solution B (90% ACN, 0.1% formic acid). The nano-HPLC was connected to a HCT Ultra high-capacity ion trap (Bruker Daltoniks, Bremen, Germany) using a PicoTip emitter (50 μm i.d., 8 μm tip i.d., New Objective, Woburn, MA, USA) and an on line nano-electrospray source. Capillary voltage was ×1.8 kV in positive mode and a dry gas flow rate of 10 L min^−1^ was used with a temperature of 180°C. The scan range used was from 300 to 1500 m/z. The mass window for precursor ion selection was ±0.2 Da and the rest of parameters were those recommended by the manufacturer for MS/MS proteomics work. Peak detection, deconvolution and processing were performed with Data Analysis 3.4 software (Bruker Daltoniks).

Protein identification was carried out using the Mascot search engine v. 2.5.0 (Matrix Science; London, UK) and the non-redundant *B. vulgaris* 20140811 (82,368 sequences; 28,127,547 residues), NCBI 20130310 (23,641,837 sequences; 8,123,359,852 residues), and Plants_EST EST_114 20140804 (158,278,518 sequences; 27,948,288,346 residues). Search parameters were: monoisotopic mass accuracy, peptide mass tolerance ±0.2 Da, fragment mass tolerance ±0.6 Da, one allowed missed cleavage, fixed modification carbamidomethylation (Cys), and variable modification oxidation (Met). Positive identification was assigned with Mascot *P*-values below the threshold (*p* < 0.05), at least two identified peptides with a score above homology, 10% sequence coverage and similar experimental and theoretical MW and pI values. We used the GO biological process annotation (http://www.geneontology.org) for classification of each individual protein identified. The secretion of apoplastic proteins was predicted using TargetP (www.cbs.dtu.dk/services/TargetP) (Emanuelsson et al., 2007), and SecretomeP (www.cbs.dtu.dk/services/SecretomeP) analysis to predict classical and non-classical secreted proteins, respectively (Bendtsen et al., 2004, 2005).

### Quantitative RT-PCR

Expression of chitinase and thaumatin genes was analyzed by qRT-PCR in two batches of plants. Total RNA from *B. vulgaris* leaves was isolated using the RNeasy Plant mini kit from QIAGEN (QIAGEN Inc., Valencia, CA, USA) according to the manufacturer's instructions. Samples were treated with DNAsa (recombinant DNase from Macherey-Nagel, Düren, Germany) to remove contaminating genomic DNA. The concentration of RNA and cDNA was determined with a Nanodrop system (Thermo Fisher Scientific, Waltham, MA, USA) and the structural integrity the RNA was checked using non-denaturing agarose gel stained with SybrSafe (Invitrogen, Carlsbad, CA, USA). One μg of total RNA was reverse transcribed to cDNA by using the SuperScript III reverse transcriptase and 2.5 μM poly(dT)_20_ primer in a final volume of 20 μl according to the manufacturer's instructions (Invitrogen, Carlsbad, CA, USA). Quantitative real time polymerase chain reactions were performed in a AB7500 Fast Real-Time PCR system (Applied Biosystems by Life Technologies, Grand Island, New York) with equal amount of cDNA for all samples and 10 μl SYBR green master mix (Applied Biosystems, Warrington, UK) using gene specific primers and two different housekeeping genes (actin and tubulin) in a final volume of 20 μl. Primer sequences and fragment sizes are listed in Table S1. The qPCR program was 50°C for 2 min, 95°C for 10 min, 40 cycles of 95°C for 15 s and 60°C for 1 min; and a final dissociation stage of 95°C for 15 s, 60°C for 1 min, and 95°C for 30 s. A previous experiment was performed to assess for primer efficiency with different sets of primers for each target gene. Primer efficiencies of the chosen sets are listed in the Table S1.

### Low-temperature scanning electron microscopy

Leaf pieces were mounted on aluminum stubs with adhesive (Gurr®, optimum cutting temperature control; BDH, Poole, UK), cryo-fixed in slush nitrogen (−196°C), cryo-transferred to a vacuum chamber at −180°C, and then fractured using a stainless steel spike. Once inside the microscope, the samples underwent superficial etching under vacuum (−90°C, 120 s, 2 kV), and then were overlaid with gold for observation and microanalysis. This freeze-fracture procedure leads to cell rupture only at the fracture plane, whereas the general internal leaf structure is well-preserved. Fractured samples were observed at low temperature with a digital scanning electron microscope (Zeiss DSM 960, Oberkochen, Germany) using secondary and back-scattered electrons. Secondary electron images (1024 × 960 pixels) were obtained at 133 eV operating at a 35° take-off angle, an accelerating voltage of 15 kV, a working distance of 25 mm and a specimen current of 1–5 nA. Microscopy was run in the Institute of Agricultural Sciences-CSIC (ICA-CSIC), Madrid, Spain.

## Results

**Figure 1:**
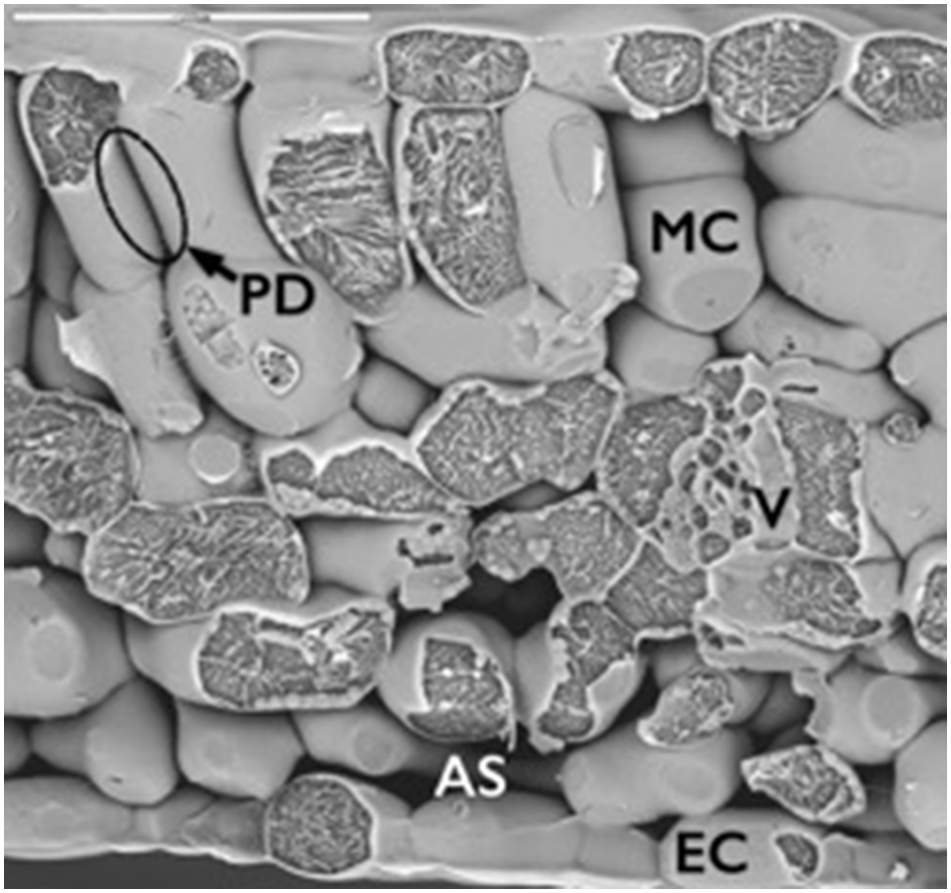
**Freeze–fracture low-temperature scanning electron micrograph of a transversal section from a *Beta vulgaris* leaf**. This leaf transversal section gives three-dimensional information on the internal structure of the leaf. Apoplastic space (AS in white letters) surrounds mesophyll (MC) and epidermal cells (EC). The image also shows the presence of plasmodesmata (PD) that communicate neighboring mesophyll cells.

Sugar beet plants showed Fe-deficiency symptoms 5 days after the treatment onset, with a marked yellowing of the younger leaves (Table S2). A freeze-fracture electron microscopy micrograph provided a representative image of a *B. vulgaris* leaf, with the apoplastic space surrounding mesophyll cells, as well as the epidermal cells and the minor vein tissues (Figure 1). The micrograph also shows the presence of plasmodesmata that communicate neighboring cells.

The apoplastic fluid collected from these leaves was assayed for c-mdh activity and only samples with contamination levels <3% (mean 1.72%, expressed on a total leaf activity basis) were used for 2-DE protein profiling (Table S2). Typical protein extraction yields ranged between 0.4 and 0.8 μg protein μL^−1^ of apoplastic fluid (Table S2).

### Protein profiles of the apoplastic fluid

**Figure 2:**
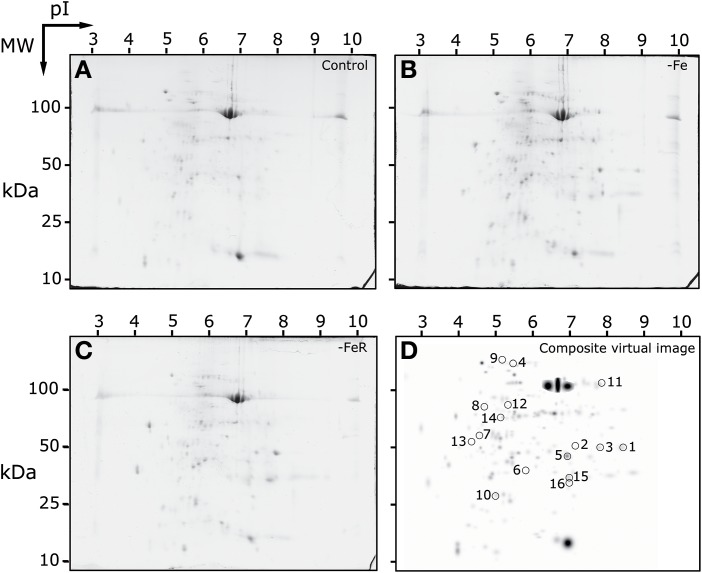
**2-DE IEF-SDS PAGE protein profile maps of leaf apoplastic fluid extracts from sugar beet plants**. Scans of typical gels from Fe-sufficient (+Fe), Fe-deficient (−Fe) and Fe-resupplied Fe-deficient (−FeR) plants are shown in **(A–C)**, respectively. To facilitate visualization of the studied spots, a virtual composite image **(D)** was created containing all spots present in the real gels **(A–C)**. In **(D)** spots whose intensities changed in relative abundance as a result of the treatments are circled and numbered as in Table 2.

The protein profile of apoplast extracts from *B. vulgaris* leaves was studied by 2-DE IEF-SDS-PAGE electrophoresis. Experimental conditions allowed for the separation of proteins within pI and MW ranges from 3.5 to 8 and from 18 to 106 kDa, respectively. Typical real scans of 2-DE gels obtained from apoplastic fluid protein extracts of Fe-sufficient (+Fe), Fe-deficient (−Fe), and Fe-resupplied Fe-deficient plants (−FeR) are shown in Figures 2A–C, respectively. The average number of detected spots (mean ± SD) was 210 ± 12, 216 ± 11, and 211 ± 20 in gels from plants grown in +Fe, −Fe, and −FeR conditions, respectively (Table S3 and Figure S1). The total number of spots consistently detected in the whole experiment (present in all four gels of at least one treatment) was 203. A composite averaged virtual map containing all consistent spots is shown in Figure 2D. All consistent spots were analyzed by MS, and proteins were unambiguously identified in 78% of the cases (158 spots) (Table S4 and Figure S1). A large percentage (97%) of identifications was achieved using the beet custom reference repository. To identify UniProt entries, BLAST searches (*E*-values < 1e-30) of the unambiguously identified protein hits were run when needed. This approach revealed a high degree of redundancy in the identified protein species. When duplicates (same UniProt entry) were removed, the 158 identified proteins were reduced to 109 non-redundant proteins and this protein set was considered as the leaf apoplastic protein profile (Table 1). However, it should be noted that there may be still certain degree of redundancy left, since some hits correspond to the same protein description but from different plant species (Table 1).

**Table 1:** **Non-redundant proteome of the leaf apoplastic fluid of sugar beet plants**.

**Number of spots^a^**	**Protein description^b^**	**UniProt^c^**	**TargetP^d^**	**SecretomeP^e^**	**Go:P^f^**
**PROTEIN METABOLISM (21)**					
2	Proteasome subunit alpha type	D7T9I6, I6U5E4	–, M	–, nCS	Ubiquitin-dependent proteolysis
1	Proteasome subunit alpha type-5	Q9M4T8	–	–	Ubiquitin-dependent proteolysis
1	Proteasome subunit alpha type-6-like isoform X1	XP_004488480	–	–	Ubiquitin-dependent proteolysis
1	Proteasome subunit beta type-6	M4D453	–	nCS	Ubiquitin-dependent proteolysis
1	Proteasome subunit alpha type-7	XP_008393990	–	–	Ubiquitin-dependent proteolysis
1	Cysteine proteinase RD19a/like	I1LJ95	S	CS	Proteolysis
1	Cysteine protease	A5HIJ1	S	CS	Proteolysis
1	Aspartic protease	A0A067FW02	S	CS	Proteolysis
2	Serine carboxypeptidase	W9SXH8, B9SMP4	S, S	CS, CS	Proteolysis
2	Serine carboxypeptidase-like 20-like	XP_008235895	S	CS	Proteolysis
2	Unknown protein with peptidase domain	E0CQB3	–	nCS	Serine-type endopeptidase activity
1	Subtilisin-like protease-like	XP_006466502	S	CS	Serine-type peptidase activity
1	Chaperonin 20	A0A061GL19	C	–	Protein folding
3	Peptidyl-prolyl cis-trans isomerase	B9RN18, O49939	C, C	–, nCS	Protein folding
1	Rubisco subunit binding-protein alpha subunit	B9MZ75	C	–	Protein folding
1	Heat shock 70 protein	O22664	–	–	Protein folding
1	Predicted heat shock cognate 70 kDa protein 2-like	XP_004505872	–	–	Protein folding
1	Elongation factor Tu	A0A067FTF8	C	nCS	Translation
**CARBON METABOLISM (27)**					
4	Enolase	Q43130	–	nCS	Glycolytic process
4	Triosephosphate isomerase	B0LT90, K4FXE7, P48496	–, –, C	–, nCS, nCS	Glycolytic process
11	Fructose-bisphosphate aldolase	F1AHC9, O04975, Q6RSN7	C, –, –	nCS, nCS, –	Glycolytic process
1	2,3-Bisphosphoglycerate-independent phosphoglycerate mutase	Q42908	–	–	Glycolytic process
1	Phosphoglucomutase	P93262	–	–	Glucose metabolic process
3	Glyceraldehyde-3-phosphate dehydogenase	A3FMH0	–	–	Glucose metabolic process
2	Ribose-5-phosphate isomerase	Q8RU73	C	nCS	PPS, non-oxidative branch
1	Ribulose-phosphate 3-epimerase	Q43157	C	nCS	Calvin cycle; PPS, oxidative branch
3	Transketolase	O20250, Q14K68	C, –	nCS, nCS	Calvin cycle; PPS, oxidative branch
1	Phosphoglycerate kinase	P29409	C	–	Calvin cycle, glycolytic process
1	Sedoheptulose-1,7-bisphosphatase	O20252	C	nCS	Calvin cycle
1	Phosphoribulokinase	P09559	C	–	Calvin cycle
6	Ribulose-1,5-bisphosphate carboxylase/oxygenase	Q08184, XP_004144069, P16032, A0A023ZPS4, Q6JXV6	C, C, C, –, –	nCS, –, nCS, nCS, nCS	Carbon fixation
3	23 kDa OEC protein	B0L802	–	–	Photosynthesis
5	Malate dehydrogenase, cytoplasmic	Q9SML8	–	nCS	Cellular carbohydrate metabolic process
5	Carbonic anhydrase	P16016, U5GK55	C, S	nCS, CS	One-carbon metabolic process
1	Oxaloacetase (*Dianthus caryophyllus*)	Q05957	–	–	Metabolic process
**STRESS AND DEFENSE (23)**					
3	Osmotin-like protein	Q38745	S	CS	Defense response
4	Thaumatin-like protein	Q6PP01, A9ZMG1, A9ZMG0, XP_004297839	–, S, S, S	nCS, CS, CS, CS	Defense response
1	Abscisic acid stress ripening-related protein	A0A059SPX5	–	–	Response to stress
1	Protein IN2-1 homolog B-like	XP_003632205	C	nCS	Glutathione metabolic process
1	Uncharacterized protein with Bet_v_I_allergen domain	M0ZYA5	–	–	Defense response
1	Ascorbate peroxidase	Q94CF7	–	–	Response to oxidative stress
1	Monodehydroascorbate reductase	Q93YG1	S	CS	Response to oxidative stress
1	Peroxidase superfamily protein	XP_007014796	S	CS	Response to oxidative stress
1	Peroxidase	P93547	S	CS	Response to oxidative stress
3	Peroxiredoxin	H6VND7	C	nCS	Cell redox homeostasis
1	Type II peroxiredoxin	I0CC96	M	nCS	Cell redox homeostasis
1	Cu/Zn superoxide dismutase	H9BQP8	C	CS	Superoxide metabolic process
6	Lactoylglutathione lyase	M0ZHD0, D2D330, Q8W593, GI:697188226, GI:697141977	C, –, C, –, C	nCS, –, nCS, –, –	Methylglyoxal catabolic process to D-lactate
2	Lactoylglutathione lyase isoform X2	GI:694332574, XP_008385524	–, –	–, –	Methylglyoxal catabolic process to D-lactate
1	Predicted isoflavone reductase homolog	XP_008377292	S	CS	Oxidation-reduction process
**POLYSACHARIDE METABOLISM (10)**					
2	3-Glucanase family protein	B9GI31	S	CS	Carbohydrate metabolic process
2	Acidic endochitinase SP2	P42820	S	CS	Polysaccharide catabolic process
1	Acidic endochitinase SE2	P36910	S	CS	Polysaccharide catabolic process
4	Chitinase	Q8LST3	S	CS	Carbohydrate metabolic process
2	Beta-xylosidase/alpha-L-arabinofuranosidase	XP_008218886	S	CS	Xylan catabolic process
1	UDP-glucuronic acid decarboxylase 1	W9R277	C	nCS	UDP-D-xylose biosynthetic process
2	Beta-fructofuranosidase	Q8VXS6, S49256	–, –	nCS, nCS	Carbohydrate metabolic process
1	Uncharacterized protein with hydrolase domain	V4SY44	M	CS	Carbohydrate metabolic process
1	Unknown protein with hydrolase domain	A9PG55	–	nCS	Mannose metabolic process
**AMINO ACID METABOLISM (7)**					
3	Serine hydroxymethyltransferase	XP_007034218, XP_007034219	–, –	–, –	L-serine metabolism
1	Serine transhydroxymethyltransferase	P50433	M	–	L-serine metabolism
1	Aminomethyltransferase	P93256	M	nCS	Glycine catabolism
3	Glutamine synthetase	Q9AWA8, Q9AXD1	C, –	–, –	Glutamine biosynthesis
2	Aspartate aminotransferase	B9HAW0	C	nCS	Cellular amino acid metabolic process
**LIPID METABOLISM (3)**					
1	3-Hydroxybutyryl-CoA dehydratase	B9RPB0	M	nCS	Enoyl-CoA hydratase activity
1	Uncharacterized protein with lipase domain	U5FE87	S	CS	Lipid metabolic process
1	Uncharacterized protein with lipase domain	D7TJU3	S	CS	Lipid metabolic process
**OTHER (9)**					
1	Ferredoxin–NADP reductase	B9SB31	C	nCS	Oxidation-reduction process
3	Alcohol dehydrogenase	B9SHB0	C	–	Oxidation-reduction process
1	Flavoprotein WrbA-like	XP_004294313	–	–	Oxidation-reduction process
2	Acylpyruvase FAHD1	XP_004508199	–	–	Hydrolase activity
1	Cytosolic ATP sulfurylase	G9B7N0	–	–	Sulfate assimilation
1	Nucleoside diphosphate kinase 2	Q01402	C	nCS	Nucleotide metabolic process
1	Thiamine thiazole synthase	XP_008244366	C	nCS	Thiamine biosynthetic process
1	Uncharacterized germin protein	I3SGS4	S	CS	Nutrient reservoir activity
1	Soluble inorganic pyrophosphatase 1	A0A061E4X1	M	nCS	Pyrophosphatase activity
**UNKNOWN FUNCTION (9)**					
1	Putative protein (*Hordeum vulgare*)	F2EID0	–	–	Nucleotide binding
4	Uncharacterized protein (*Vitis vinifera*)	D7SXW6	S	CS	
1	Uncharacterized protein (*Jatropha curcas*)	A0A067KSH6	M	nCS	
1	Uncharacterized protein (*Jatropha curcas*)	A0A067KHD5	–	nCS	
1	Jasmonate-induced protein (*Atriplex canescens*)	P42764	–	nCS	
1	CSP41A protein	E5KGE2	–	–	Cellular metabolic process
1	Hypothetical protein CICLE_v10029208mg (*Citrus clementina*)	V4SBG5	S	CS	
2	No blast result				
**NO IDENTIFIED**					
45					

a *Number of spots with the same protein description*.

b *Protein description*.

c *UniProt entries sharing same protein description*.

d *TargetP algorithm predictions: C, M, S, and—indicate chloroplast, mitochondrion, secretory pathway and any other location, respectively*.

e *SecretomeP algorithm predictions: CS, nCS and—indicate classical secreted, non-classical secreted, and non-secreted proteins, respectively*.

f *Description of the GO: P (biological process) term*.

**Figure 3:**
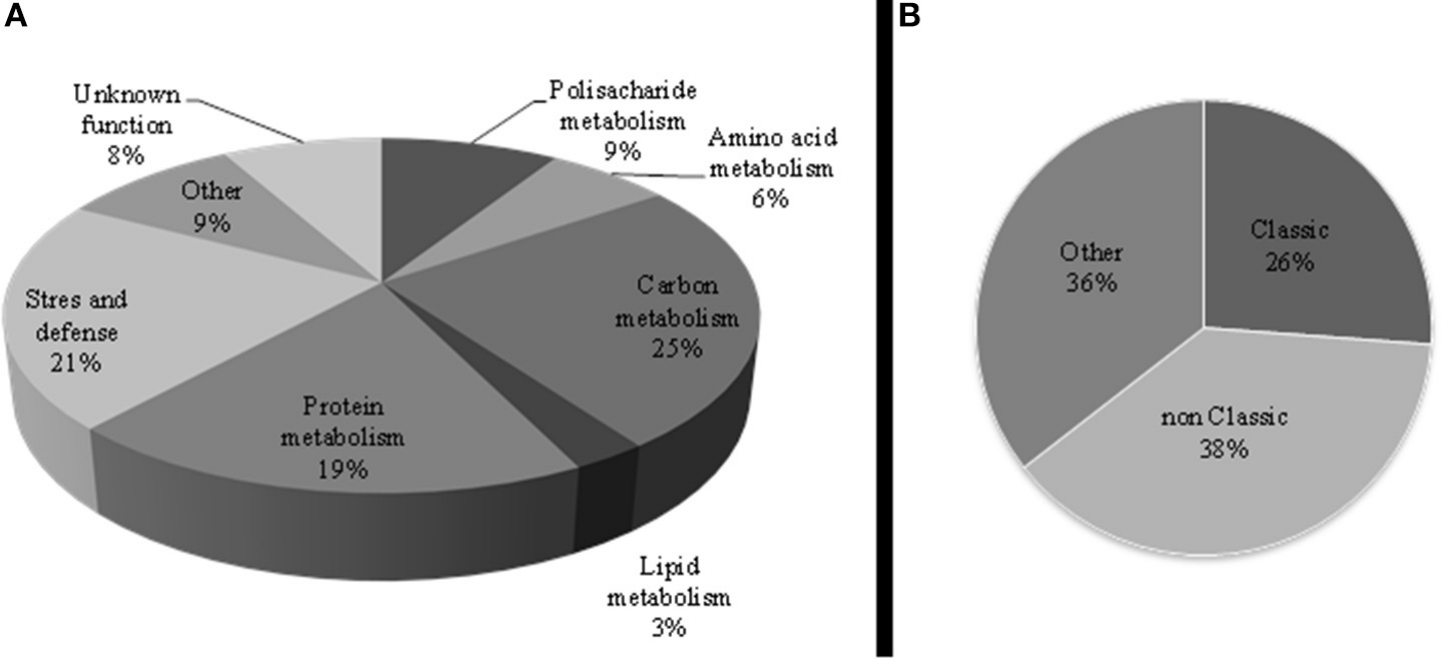
**(A)** Functional classification of the non-redundant leaf apoplastic proteome of sugar beet. Pathways related to the identified proteins were integrated according to the GO annotation. **(B)** Protein distribution of the non-redundant leaf apoplastic proteome according to TargetP and SecretomeP algorithms; CS and nCS indicate classical and non-classical secretory proteins, respectively, whereas “Other” represents proteins ascribed to other cellular compartments and unclassified.

The distribution of non-redundant proteins according to the biological process in the sugar beet leaf apoplast indicated that the major functional categories within the apoplastic proteome were C metabolism (25%; 27 protein species), stress and defense (21%; 23 proteins), and protein related processes (19%; 21 proteins), followed by cell wall related processes (9%, 10 proteins) (Table 1, Figure 3A).

From the non-redundant leaf apoplastic proteins, 26% (28 protein species) were predicted to have a signal peptide sequence using the TargetP or SecretomeP softwares, whereas SecretomeP revealed that 38% (41 proteins) were assigned to non-classical secreted proteins lacking a signal peptide (Table 1, Figure 3B).

### Effect of Fe-deficiency and Fe resupply on the leaf apoplastic fluid protein profile

**Figure 4:**
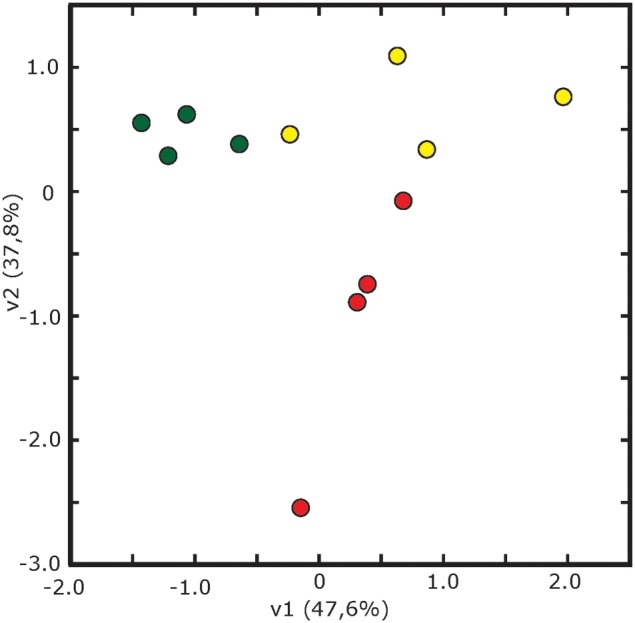
**Multivariate statistical analysis (Principal Component Analysis, PCA) of 2-DE gels**. Score scatter PCA plot of component 1 vs. component 2 after analysis of spots showing differences in relative abundance from leaf apoplastic fluid of sugar beet plants grown in Fe-sufficient (green circles), Fe-deficient (yellow circles) and Fe-resupplied Fe-deficient (red circles) conditions.

The statistical analysis (*p* < 0.05; *t*-Student) of averaged 2-DE maps indicated that 8% (16 spots) of the total number of consistent spots changed significantly and >2-fold in relative abundance in the whole experiment (spots labeled 1–16 in Figure 2D). Among them, 88% of the spots (14) matched reliably to known proteins and were identified by database searches (1–14, Figure 2D). Their metabolic functions were assessed manually by GO annotation and identified proteins were classified into five functional categories: cell wall related processes (4 spots, 2 proteins), protein metabolism (4 proteins), and defense, amino acid and C-related pathways, with two proteins each (Table 2). The principal component analysis of differentially accumulated spots showed a good separation between treatments, with the first and second components explaining approximately 48 and 38% of the variation, respectively (Figure 4).

**Table 2:** **Spots showing differences in relative abundance (Student *t*-test *p* < 0.05) as a result of Fe deficiency and Fe resupply**.

**Spot number in Figure 1^a^**	**SSP number^b^**	**−Fe vs. +Fe^c^**	**−FeR vs. +Fe^d^**	**−FeR vs. −Fe^e^**	**Protein^f^**	**Plant Species^g^**	**Uniprot^h^**	**GO:P^i^**	**SecretomeP^j^**
**POLYSACHARIDE CATABOLISM**									
1	9403	***new***	*new*	−8.1	Chitinase from *Phytolacca americana*	*Beta vulgaris*	Q8LST3	Carbohydrate metabolic process	CS
2	7404	**8.7**	**5.3**	−1.6	Chitinase from *Phytolacca americana*	*Beta vulgaris*	Q8LST3	Carbohydrate metabolic process	CS
3	8401	**4.2**	3.0	−1.4	Chitinase from *Phytolacca americana*	*Beta vulgaris*	Q8LST3	Carbohydrate metabolic process	CS
4	3805	1.4	−3.6	−**5.0**	Beta-xylosidase/alpha-L-arabinofuranosidase from *Prunus mume*	*Beta vulgaris*	XP_008218886^*^	Xylan catabolic process	CS
**PROTEIN RELATED**									
5	1402	−1.8	−**6.5**	−3.7	Cysteine proteinase RD19a-like from *Glycine max*	*Beta vulgaris*	I1LJ95	Proteolysis	CS
6	2203	−1.1	−**3.6**	−3.2	Peptidyl-prolyl cis-trans isomerase from *Ricinus communis*	*Beta vulgaris*	B9RN18	Protein folding	nCS
7	1602	−3.1	−**7.9**	−2.6	Peptidyl-prolyl cis-trans isomerase from *Spinacia oleracea*	*Beta vulgaris*	O49939	Protein folding	nCS
8	2902	1.2	−2.1	−**2.4**	Cytosolic heat shock 70 protein from *Spinacia oleracea*	*Beta vulgaris*	O22664	Protein folding	–
**DEFENSE**									
9	1404	**19.5**	**11.5**	−1.7	Thaumatin-like protein 1 from *Fragaria vesca*	*Beta vulgaris*	XP_004297839^*^	Defense response	CS
10	2503	1.2	−2.0	−**2.3**	Lactoylglutathione lyase from *Gossypium hirsutum*	*Beta vulgaris*	D2D330	Methylglyoxal catabolic process to D-lactate	nCS
**AMINO ACID METABOLISM**									
11	8710	−1.7	−**5.0**	−3.0	Serine hydroxymethyltransferase from *Theobroma cacao*	*Beta vulgaris*	XP_007034218^*^	L-serine metabolic process	–
12	2610	2.3	−6.3	−**14.2**	Glutamine synthetase	*Beta vulgaris*	Q9AXD1	Glutamine biosynthesis	–
**CARBON METABOLISM**									
13	7302	−**2.2**	−1.8	1.2	Carbonic anhydrase from *Spinacia oleracea*	*Beta vulgaris*	P16016	One-carbon metabolic process	nCS
14	4307	ND	−12.2	***new***	23 kDa OEC protein from *Salicornia veneta*	*Beta vulgaris*	B0L802	Photosynthesis	–
**NO IDENTIFIED**									
15	7202	1.5	**6.1**	**4.1**					
16	7204	4.7	**2.4**	−2.0					

a *Spot number as in Figure 1*.

b *Spot number as in Table S3*.

c–e *Fold change in the Fe-deficient vs. Fe sufficient, Fe resupplied vs. Fe-sufficient and Fe-resupplied vs. Fe-deficient comparisons, respectively, values in bold indicate significant changes and when the ratios were lower than one the inverse was taken and the sign changed*.

f *Protein description*.

g *Plant Species*.

h Uniprot entry (^*^denotes protein entry in UniParc).

i *GO:P term description*.

j *SecretomeP algorithm predictions: CS, nCS and—indicate classical secreted, non-classical secreted and non-secreted proteins, respectively*.

When the protein profile of -Fe samples was compared to that of the +Fe samples, only five spots changed in relative abundance. Four of them increased, including three spots (spots 1–3) identified as chitinase and one (spot 9) identified as a thaumatin-like protein. Only one spot (spot 13) decreased as a result of Fe deficiency and it was identified as carbonic anhydrase (Figure 2D, Table 2).

In the comparison of −FeR vs. +Fe, eight spots changed in relative abundance, four of them increased whereas the other four decreased (Figure 2D, Table 2). Two of the spots increasing in relative abundance were identified as chitinase and thaumatin (spots 2 and 9, respectively) whereas the other two could not be identified (spots 15 and 16). The spots decreasing in relative abundance were identified as a cysteine proteinase RD19-like protein (spot 5), a peptidyl-prolyl *cis*-*trans* isomerase (spots 6, 7) and a serine hydroxymethyltransferase (spot 11) (Table 2). When the -FeR samples were compared to the –Fe ones, six spots changed in relative abundance (Table 2). Among them, one spot was detected *de novo* (spot 14) and identified as the 23 kDa OEC protein and a second one (spot 15) could not be identified. Four spots showed significant decreases in relative abundance (Table 2, Figure 2D), and they were identified as β-xylosidase/alpha-L-arabinofuranosidase (spot 4), a cytosolic heat shock 70 protein (spot 8), lactoylglutathione lyase (spot 10) and glutamine synthetase (spot 12).

### Relative transcript abundance of target genes

**Figure 5:**
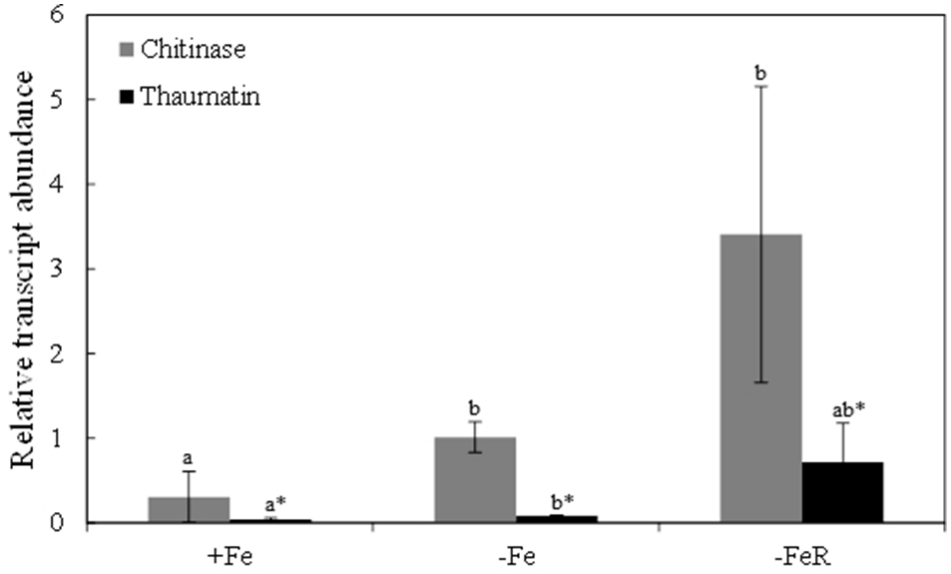
**Relative abundances of chitinase and thaumatin-like 1 transcripts measured in leaves by qRT-PCR using tubulin as housekeeper gene**. Sequences containing the peptides matched during protein identification, KDHBv_S14175_58500.t1 for chitinase and BQ584258 for the thaumatin-like 1, were selected to design primers for specific amplification (Table S1). Data are means ± SD of two experiments with four biological and two technical replicates per treatment in each experiment. Different letters indicate statistically significant differences at *p* < 0.05.

The chitinase and thaumatin-like 1 proteins identified in spots 1 and 9 (Table 2 and Table S4, Figure 2), respectively, showed the highest increases in relative abundance as a result of Fe-deficiency and were selected to study transcript abundances using q-PCR. Sequences containing the peptides matched during protein identification, KDHBv_S14175_58500.t1 for chitinase and BQ584258 for the thaumatin-like 1 protein, were selected to design primers for specific amplification of target genes (Table S1). In Fe-deficient leaves, the relative abundances of chitinase and thaumatin-like 1 protein transcripts increased 3- and 2-fold, respectively, when compared to controls (Figure 5), whereas upon Fe resupply the relative abundance of chitinase transcript was higher and that of thaumatin-like 1 protein was not significantly different at *p* < 0.05 (Figure 5). When compared to the Fe-deficient controls, changes in transcript abundances upon Fe-resupply were not statistically significant at *p* < 0.05 (Figure 5).

## Discussion

### Leaf apoplastic protein profile

The 2-DE proteomic approach allowed us to resolve 203 spots, with 158 of them (78%) being identified and 109 accounting for non-redundant proteins. These results are similar to those reported for gel-based leaf apoplastic proteome studies in other plant species, which ranged between 93 and 470 spots, with an average of 200 spots in most of the studies (Table 3 and references therein). Functional classification of the non-redundant leaf apoplastic proteins of sugar beet indicates that stress and defense, protein metabolism, cell wall and C metabolism account for approximately 75% of the identified proteome (Figure 3A).

**Table 3:** **Summary of proteomic studies on leaf apoplast**.

**References**	**Plant specie**	**Technique**	**Leaf material**	**Study**	**Separation**		**Number of spots**	**Analysis**	**Changes**	**Functional classification in control**
Fecht-Christoffers et al., 2003	*Vigna unguiculata*	VIC-(water)	Whole leaf	Mn toxicity	18 cm	3–10 N	Not reported		10	Not reported
					18 cm	BN-PAGE				
Boudart et al., 2005	*Arabidopsis thaliana*	VIC-(0.3 M mannitol)	Whole rossete leaves	Mapping	7 cm	4–7	46	93 identified	–	32 % cell wall modifying proteins, 21 % defense-related, 20% proteins with interacting domains, 11 % proteinases
		VIC-(1 M NaCl, 0.2 M CaCl_2_, 2 M LiCL or 0.3 M CDTA)	Whole rossete leaves		Sepharose fractioning followed by 1DE		87		–	
Dani et al., 2005	*Nicotiana tabacum*	VIC- (50 mM phosphate buffer, 200mM NaCl, pH 7)	1-2 cm 2 leaf strips	Salt stress	7 cm	3–10	150		20	Not reported
Soares et al., 2007	*Medicago truncatula*	VIC-(15 mM sodium acetate buffer, pH 4.5)	Leaf pices	Mapping	Not specified	3–10 NL	220	45 analized and 19 identified	–	38% defense, 58 % unknown
Casasoli et al., 2008	*Arabidopsis thaliana*	VIC-(25 mM Tris-HCl pH7.4, 50mM EDTA, 150 mM MgCl_2_)	Seedlings	Oligogalacturonides	DIGE-13 cm	3–10	180–220	62 analyzed and 55 identified	16	30% secretory pathway, 20% chloroplast, 14% cell wall
Floerl et al., 2008	*Brassica napus*	VIC-(100 mM KCl, 0.01% Triton)	Leaf without midrib	Fungus infection	18 cm	3–10	170	31 analyzed and 19 identified	12	Chitinases, proteases, peroxidases, glucanases, germin, cell wall modification
Soares et al., 2009	*Medicago truncatula*	VIC-(water)	Leaf-halves	Wounding	Not specified	3–10 NL	110	35 analized and identified	28	Defense or unknown
Pechanova et al., 2010	*Populus deltoides*	Pressure chamber	Whole leaf	Mapping	24 cm	3–10 NL	84	144 analyzed and identified	–	25% cell wall, 24% stress-defense, 12% proteolysis, 8% cell wall, 4% carbohydrate
					2D-LC		106		–	
Goulet et al., 2010	*Nicotiana benthamiana*	VIC (10mM MES, pH 5.8)	Whole leaf	Agrobacterium infection	13cm	3–10 NL	Ca. 200	91 analyzed and 73 identified	44	29% defense, 15% cell wall, 6% proteases
Witzel et al., 2011	*Zea mays*	VIC-(water/ 100mM sodium phosphate buffer/25 mM Tris-Hcl/ 100 mM sorbitol/20 mM ascorbic acid and 20 mM CaCl_2_/ 50 mM NaCll)	5.5 cm leaf segments	Infiltration technique	7 cm	3–10	171/107/131/133/133/114 328 total	67 identified as apoplastic proteins by in silico (20%)	–	58% Carbohydrate metabolism and cell wall, 22% defense, 10% protein transporting function, 27% cytosol proteins, 13% chloroplast proteins
Floerl et al., 2012	*Arabidopsis thaliana*	VIC (100 mM KCl, 0.005% Triton X-100	Whole leaf	Fungus infection	18 cm	3–10	217	45 analyzed and identified	7	25% carbohydrate metabolism, 18% proteases, 21% oxidoreductases
Shenton et al., 2012	*Oryza sativa*	VIC-(50 mM Na-P buffer pH7.5, 600 mM NaCl, 0.01% Tween-20, 0.1% β-mercapto ethanol)	Whole leaf	Fungus infection	11 cm	3–11 NL	Not reported		–	Not reported
Delaunois et al., 2013	*Vitis vinifera*	VIC-(150 mM Tris-HCl, pH 8.5, 6mM CHAPS)	1 cm 2 leaf cuts	Mapping	18 cm	3–10 NL	306	177 analyzed and 89 identified	–	On protein number basis: 28% defense, 18% cell wall / on spot volume basis: 51% defense, 16% proteases, 12% cell wall
Kim et al., 2013	*Oryza sativa*	CA-VIC (200 mM CaCl_2_, 5mM Na-acetate, pH 4.3)	5 cm leaf cuts	Fungus infection	18 cm	4-7	27		12	21% carbohydrate metabolism, 20 % protein metabolism, 30 % defense, 12 % energy pathway
					MudPIT		283		–	
Petriccione et al., 2014	*Actinidia deliciosa*	VIC-(100mM Tris-HCl, pH 7.5, 10 mM KCl, 1mM phenylmethanesulfonyl fluoride)	Leaf without midrib	Pseudomonas infection	7 cm	3-10	ca. 220		60	Not reported
Kim et al., 2014	*Oryza sativa*	CA-VIC-(200 mM CaCl_2_, 5 mM Na-acetate, pH 4.3)	5 cm leaf cuts	Fungus infection	Shotgun		470	174 secreted proteins by in silico analyses		Not reported

*VIC stands for vacuum infiltration followed by centrifugation*.

Stress and defense related proteins accounted for 21% of the non-redundant sugar beet apoplastic proteome. Similar to what has been reported in other plant species (references in Table 3), proteins identified were peroxidases, osmotin-like, thaumatin-like, and pathogenesis-related proteins. Our results also indicate the presence in the leaf apoplast of enzymes participating in defense against oxidative stress (two peroxiredoxins, CuZnSOD and two enzymes from the ascorbate-glutathione cycle: ascorbate peroxidase and monodehydroascorbate reductase) and in the detoxification of methylglyoxal (two UniProt entries described as glyoxalase I). These proteins have also been found in the leaf apoplast from poplar (Pechanova et al., 2010) and are also present in fluids from the vascular tissue (Lattanzio et al., 2013; Lucas et al., 2013). The presence of this wide spectrum of defense proteins in non-stressed plants has been attributed to a pre-formed defense that creates a hostile environment for pathogens (Pechanova et al., 2010; Delaunois et al., 2013).

The contribution of protein metabolism-related proteins to the sugar beet apoplastic proteome (19%, with 14 of the 21 UniProt entries being proteases) is within the range reported in other studies using grapevine, Arabidopsis, and rice (16–20%; Floerl et al., 2012; Delaunois et al., 2013; Kim et al., 2013), but higher than percentages described for other plant species, which range from 6 to 10% (Boudart et al., 2005; Goulet et al., 2010; Pechanova et al., 2010). The presence of such a high number of proteolysis-related proteins in the apoplast has been proposed to be species-dependent (Delaunois et al., 2013). Subtilisin-like, serine-carboxypeptidases, and aspartic proteases found in this study have been consistently described in the apoplast of several plant species (Goulet et al., 2010; Floerl et al., 2012; Delaunois et al., 2013). In addition, our results indicate that sugar beet apoplast also contains several subunits of the proteasome (six UniProt entries), which is involved in the ubiquitin dependent degradation of damaged and miss-folded proteins (Kurepa and Smalle, 2008). These apoplastic proteases may play a role in plant defense against pathogens and also in signaling (Van Der Hoorn and Jones, 2004; Xia et al., 2004; Pearce et al., 2010).

The cell wall related category accounted for 9% (10 proteins) of the sugar beet leaf proteome and included nine glycoside hydrolases. Glycoside hydrolases modify cell walls by metabolizing carbohydrate compounds from plant cell polysaccharides and by interacting with hemicellulases and pectic enzymes (Numan and Bhosle, 2006; Minic, 2008). The percentage of cell wall related proteins found in sugar beet is similar to those reported in grapevine and tobacco but lower than those found in rice, poplar or maize (Table 3). On the other hand, the percentage of proteins participating in C-related processes in the leaf apoplast of sugar beet was slightly higher (25%, 27 proteins) than those reported in other plant species (Table 3 and references therein). However, these values vary depending on the functional classification of certain proteins, such as peroxidases that are included in defense or cell wall depending on the study, and on the consideration of carbohydrate metabolism and cell wall as one or two functional categories.

The relatively high percentage of C-related proteins found in the sugar beet leaf proteome may have several causes. First, a large number of the spots identified as proteins participating in C metabolism have a low spot intensity and therefore they are over-represented in the functional categorization based on protein number. Second, the sugar beet apoplastic proteome was obtained by direct leaf centrifugation, whereas most of the proteomes from other plant species were obtained using leaf vacuum infiltration followed by centrifugation (Table 3 and references therein); this later technique may be somewhat better at preventing leakage of proteins from the cytoplasm (Lohaus et al., 2001; Witzel et al., 2011). Interestingly, the percentage of C-related proteins in the stem apoplast of poplar (18%) as well as the identity (TCA and glycolysis-related) (Pechanova et al., 2010), are similar to those found in sugar beet (25%). This comparison might suggest that the sugar beet apoplastic fluid obtained by direct centrifugation of the leaves could have a high contribution of the fluid contained in the xylem sap vessels of the main vein of the leaf.

The presence of cytoplasmic contamination in our samples was always lower than 3%, with an average value of 1.5%, as assessed by the activity of c-mdh (Table S2). However, *in silico* analysis of the non-redundant proteome using the TargetP and the Secretome P algorithms (for classical and non-classical secretory proteins) predicted 26% (28 proteins) and 38% (41 proteins) of the non-redundant proteome as classical and non-classical secretory proteins, respectively, whereas 36% of the proteins were predicted to be non-secretory (Table 1, Figure 3B). This percentage (64%) of secretory proteins is within the range of those reported in the leaf apoplast proteome of other plant species (from 50% in Arabidopsis to 80% in grapevine and poplar; Casasoli et al., 2008; Pechanova et al., 2010; Delaunois et al., 2013). The fact that our samples contain a relative large number of proteins tagged as non-secretory is not surprising, since the c-mdh assay indicates that up to 3% of the leaf cells could have delivered cytosolic components into the isolated apoplastic fluid. Furthermore, the release of cytosolic components may be in part associated to the rupture of the pasmodesmata that exist in these leaves (Figure 1), even if mesophyll cells remain intact. On the other hand, RuBisCO was identified in six spots of the total 203 of the leaf apoplastic proteome map (Table S4). In addition there were 10 more spots whose identification yielded proteins related to the Calvin cycle and are, most likely, plastid located (Table 1). Therefore, we could assume that at least 16 spots (approximately 8% of the total) are probably a result of cell leakage. These results may suggest that the real contamination by cell rupture is likely to be higher than that estimated by the use of c-mdh as a contamination marker.

#### Changes induced by Fe deficiency in the leaf apoplastic proteome

The largest part of the changes caused by Fe-deficiency and Fe-resupply corresponded to proteins tagged as secretory proteins (10 spots), probably corresponding to true components of the apoplast. Changes also occurred in a relatively small number (four) of the apoplastic fluid proteins tagged as non-secretory ones, and possibly associated to cell or plasmodesmata rupture (these are marked by ^*^ in the following paragraphs).

Iron-deficiency caused changes in the relative abundance of five spots (2.5% of the consistent spots of the leaf apoplast proteome), suggesting that protein homeostasis in the leaf apoplast fluid is well-maintained upon Fe shortage. This number of changes is markedly low when compared to those induced by Fe deficiency in other proteomes, including those of roots and thylakoids of sugar beet plants (44 and 53%, respectively; Andaluz et al., 2006; Rellán-Álvarez et al., 2010) and falls within the lower range of the number of changes caused by other abiotic stresses in the leaf apoplast (Table 3; Dani et al., 2005; Casasoli et al., 2008).

Three spots identified as chitinase increased in relative abundance as a result of Fe-deficiency (spots 1–3; Figure 2, Table 2). These three spots had the same molecular weight and different pIs (8.2, 7.1, and 7.6), indicating that Fe-deficiency alters the isoform pattern of this enzyme, with one of the isoforms identified *de novo* in Fe-deficient samples. Chitinases are hydrolytic enzymes that break down glycosidic bonds, removing xylosyl residues of xyloglucan oligosaccharides in the cell wall (Sampedro et al., 2001). Therefore, these increases in chitinase suggest the existence of Fe deficiency-induced cell wall modifications. This is in agreement with the changes elicited by Fe deficiency in leaf morphology, which include reduction of xylem vessel size (Fernández et al., 2008; Eichert et al., 2010). Furthermore, changes in lignification have been reported in roots of Fe-deficient pear and quince cultivars (Donnini et al., 2009) and cell wall related proteins commonly show changes in abundance in proteomic studies of Fe deficient plants (see López-Millán et al., 2013 for a review).

A spot identified as a thaumatin-like protein 1 (spot 9) also increased in relative abundance as a result of Fe-deficiency. Thaumathins are pathogenesis-related (PR) proteins from the PR5 subfamily, which are induced by biotic and abiotic stresses. Some members of PR5 subfamily have been described to play distinctive roles in the defense system that protects against high-salt stress or osmotic imbalance (Tachi et al., 2009), which is likely to occur in the Fe-deficiency treatment in the presence of CaCO_3_. A PR5b protein also showed increases in abundance in roots and stems of Fe-deficient *M. truncatula* plants grown in the presence of CaCO_3_ (Rodríguez-Celma et al., 2011), suggesting that thaumatins are ubiquitously up-regulated by Fe deficiency. Interestingly, both chitinase and the thaumatin-like 1 protein were also affected in the leaf proteome of cowpea submitted to Mn toxicity (Fecht-Christoffers et al., 2003).

Only one protein, identified as carbonic anhydrase (CA; spot 13), decreased as a result of Fe deficiency. Carbonic anhydrase interconverts CO_2_ and bicarbonate to maintain the acid-base balance. A decrease in CA activity could be attributed to the presence of bicarbonate in the nutrient solution that may reach the leaf apoplast *via* xylem (Nikolic and Römheld, 2007). Although some mammalian CA isoforms are extra-cellular and have been described in saliva and milk (Karhumaa et al., 2001; Leinonen et al., 2001), and CA is classified as a non-classical secretory protein by SecretomeP, plant isoforms are distinct from an evolutionary standpoint and have been mainly localized in the chloroplast or the cytosol.

#### Changes induced by Fe resupply in the leaf apoplastic proteome

Iron resupply to Fe deficient plants caused significant changes in the relative abundance of 13 spots when compared to either Fe-sufficient or Fe-deficient plants. These spots can be roughly classified into two major groups. The largest group (seven spots) was composed by those spots increasing in relative abundance (significantly or not) with Fe-deficiency and decreasing significantly with Fe resupply when compared either with the control or with Fe-deficient samples. This group contained two cell wall related proteins [chitinase (spot 2), and β-xylosidase (spot 4)], three stress-related proteins [a heat shock 70 protein (spot 8^*^), thaumatin-like 1 protein (spot 9), glyoxalase I (spot 10)], glutamine synthase (spot 12^*^) and the unidentified spot 16. These results indicate that Fe resupply causes changes in the short-term (within 24 h) in cell wall and stress-related processes of the Fe-resupplied plants toward values found in the Fe-sufficient controls. One more protein (spot 14^*^, the 23 kDa OEC protein, which nuclear-encoded and synthesized in the cytosol) was also responsive to short term Fe-resupply but followed a different trend, not-detected in Fe-deficient samples but detected upon Fe-resupply. This likely reflects transitory increases in the cytosolic levels of this protein upon short term Fe-resupply, which are necessary for the slight recovery of the photosynthetic system at this resupply stage (Larbi et al., 2004).

On the other hand, a second group of four spots (spots 5–7 and 11^*^) decreased in relative abundance with Fe deficiency (although not significantly) and decreased significantly upon Fe-resupply when compared either with controls or Fe-deficient samples. This group included two proteins identified as protein-metabolism related [a cysteine protease (spot 5), a peptidyl-prolyl *cis*-trans isomerase (spots 6,7)] and a serine hydroxymethyltransferase (spot 11^*^). One more spot (spot 15, unidentified protein) followed the opposite behavior (increased). This group of proteins can be classified as not responsive to short term Fe-resupply, since they may require a time longer than 24 h to reset to control values after resupply.

## Concluding remarks

In summary, this study provides information on the composition of the apoplast proteome in *B. vulgaris* leaves, which appears to be quite similar to that of other previously studied plant species. The study shows that Fe deficiency and Fe resupply cause significant changes in a limited number of proteins in the leaf apoplast, and none of them is expected to play a significant role in metal homeostasis. This is in contrast with the intense changes previously found in the concentrations of metabolites (e.g., carboxylates) that could interact with metals in the same compartment. Data found contribute toward the understanding of metal homeostasis, and in particular on the still poorly known mechanisms of Fe acquisition by plant mesophyll cells. Results presented here open an interesting line of work regarding possible modifications of cell wall that ultimately may affect permeability or transport of Fe across the plasma membrane.

### Conflict of interest statement

The authors declare that the research was conducted in the absence of any commercial or financial relationships that could be construed as a potential conflict of interest.

## Supplementary material

Figure S1 **Virtual composite image containing all consistent spots present in the real gels**. Numbers are as in Table S4 (SSP).
