# Supplementary figures and images for: Protein profile of Beta vulgaris leaf apoplastic fluid and changes induced by Fe deficiency and Fe resupply
Source: Front Plant Sci. 2015 Mar 18;6:145. doi: 10.3389/fpls.2015.00145 (PMC4364163; doi:10.3389/fpls.2015.00145)

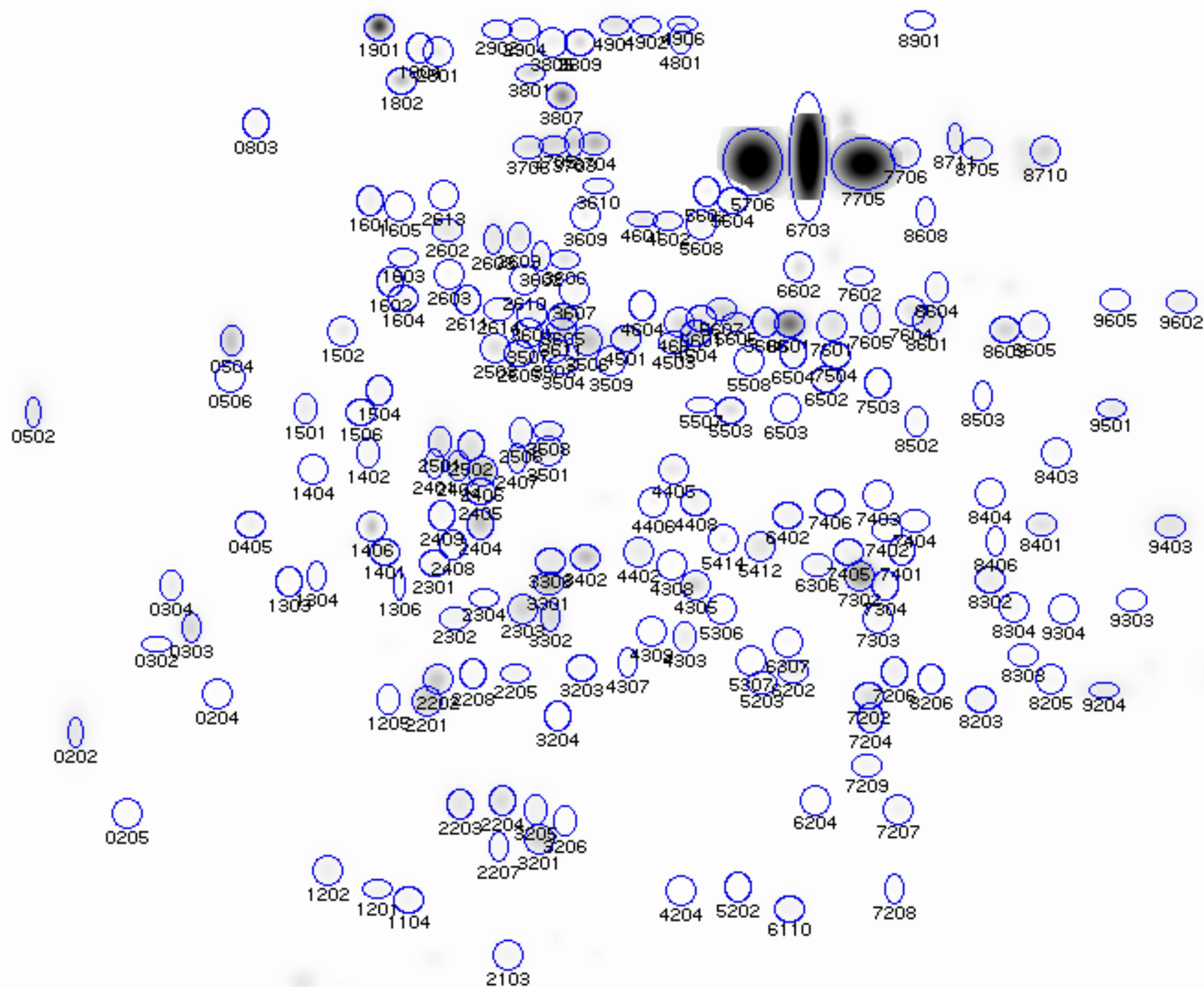

Supplement: Figure S1 — Virtual composite image containing all consistent spots present in the real gels. Numbers are as in Table S4 (SSP). [file Image1.PDF]
