# Supplementary material for: Protein profile of Beta vulgaris leaf apoplastic fluid and changes induced by Fe deficiency and Fe resupply
Source: Front Plant Sci. 2015 Mar 18;6:145. doi: 10.3389/fpls.2015.00145 (PMC4364163; doi:10.3389/fpls.2015.00145)
Supplement: Supplementary file 2 [file Table1.PDF]

Table S1. Forward and reverse primers used for qPCR analyses and primer set efficiencies.

Gene name	Entry	Fordward primer	Reverse primer	Size (bp)	Primer set efficiency
Actin	HQ656028.1	ATCACGACCAGCAAGATCCA	TGCTTGACTCTGGTGATGGT	20	0.98
Tubulin	*KDHBv_S03172_148850.t1*	TAGCCCAGTTGTTACCAGCA	GATGGATCTTGAGCCGGGTA	20	0.91
Chitinase	*KDHBv_S14175_58500.t1*	TTGCTAGTGCAGCGTAGTGG	TCTATTGGTGGTGGTGCAGA	20	0.88
Thaumatin	BQ584258	ATCTCGCCAGAACCACAATC	CCATATGGCCAGGGACTCTA	20	0.91
