# Supplementary material for: Protein profile of Beta vulgaris leaf apoplastic fluid and changes induced by Fe deficiency and Fe resupply
Source: Front Plant Sci. 2015 Mar 18;6:145. doi: 10.3389/fpls.2015.00145 (PMC4364163; doi:10.3389/fpls.2015.00145)
Supplement: Supplementary file 3 [file Table2.PDF]

Table S2. Summary of the 2-DE protein profiling results. SPAD values, apoplastic volume collected (µL $leaf^{-1}$), leaf homogenate c-mdh activity (nmol g^{-1} FW s^{-1}), apoplastic c-mdh activity (nmol mL^{-1} s^{-1}), percentaje of c-mdh activity on total leaf c-mdh activity basis, protein yield (µg protein $µL^{-1}$ apoplastic fluid), average number of spots, number of spots changing significantly in relative abundance (t-Student test at $p<0.05$), and number of spots newly detected, showing increased and decreased relative abundance. Numbers in brackets indicate identified proteins.

	**+Fe**	**-Fe**	**-FeR**
Average leaf SPAD value	30.5 ± 2.5	6.7 ± 0.5	
Apoplastic fluid volume (µL/leaf)	144 ± 38	134 ± 37	158 ± 42
Leaf homogenate mdh activity (nmol g^{-1} FW s^{-1})	472 ± 200	626 ± 321	627 ± 256
Apoplastic c-mdh activity (nmol mL^{-1} s^{-1})	8.3± 4.0	7.6 ± 4.5	10.4 ± 5.4
% c-mdh in apoplast	1.7 ± 0.9	1.2 ± 0.7	1.7 ± 0.9
Protein yield (µg protein $µL^{-1}$)	0.43 ± 0.31	0.58 ± 0.47	0.78 ± 0.30
Number of spots	210 ± 12	216 ± 11	211 ± 20
Number of consistent spots		203	
	**-Fe *vs.* +Fe**	**-FeR vs. +Fe**	**-FeR vs. -Fe**
Spots with significant intensity changes	5	8	6
Number of new spots	1 (1)	0	1 (1)
Number of spots with increased abundance	3 (3)	4 (2)	1 (0)
Number of spots with decreased abundance	1 (1)	4 (4)	4 (4)
