# Supplementary material for: Protein profile of Beta vulgaris leaf apoplastic fluid and changes induced by Fe deficiency and Fe resupply
Source: Front Plant Sci. 2015 Mar 18;6:145. doi: 10.3389/fpls.2015.00145 (PMC4364163; doi:10.3389/fpls.2015.00145)
Supplement: Supplementary file 4 [file Table3.PDF]

Table S3. Univariate statistical analysis of 2-DE gels. Spot volumes in all samples (12 gels, 3 treatments and 4 biological replicates) are listec in columns 2-13. (a) spot number as in Fig S1 and Table S4; (b-d) p values (t-Student test) of Fe-deficient *vs.* Fe-sufficient, Fe-resupplied *vs.* Fe-sufficient and Fe-resupplied *vs.* Fe-deficient comparisons, respectively.

SSP[a]	C1	C2	C3	C4	-Fe1	-Fe2	-Fe3	-Fe4	-FeR1	-FeR2	-FeR3	-FeR4	Mean (+Fe)	Mean (-Fe)	Mean (-FeR)	ratio -Fe/+Fe	p -Fe vs +Fe[b]	ratio -FeR/+Fe	p -FeR vs +Fe[c]	ratio -Fe/-FeR	p -Fe vs -FeR[d]
202	7459.1	14668.6	5304.7	0	2.5	286.2	0.7	2.2	1249.5	656.7	51.4	1.5	6858.1	72.9	489.8	0.01	0.11	0.07	0.11	0.15	0.24
204	439.1	0	30.9	10.7	2373.1	1630.9	272.2	840.3	1475	0	2497.4	313.9	120.2	1279.1	1071.6	10.64	0.06	8.92	0.18	1.19	0.82
205	885.4	824.3	343.8	6.6	4.4	3.9	1.9	5.5	15.7	0	3	0	515.0	3.9	4.7	0.01	0.09	0.01	0.09	0.84	0.86
302	6134.1	8812	148.6	0	23126.9	15334	5736.8	126969	13440.3	51585.5	27815.8	15654.7	3773.7	42791.6	27124.1	11.34	0.28	7.19	0.06	1.58	0.67
303	48.1	0	0	0	2370.9	2182.7	1632.8	14349	3691.7	468.5	0	101.5	12.0	5133.9	1065.4	426.93	0.19	88.60	0.31	4.82	0.33
304	212.1	0	0	0	297.7	5545.4	1.6	720.2	187.4	986.6	1731.5	283.4	53.0	1641.2	797.2	30.95	0.32	15.03	0.15	2.06	0.57
405	4116	2008.9	765.5	741.9	469	857.6	1017.8	865.7	1143.6	1356.8	62	51	1908.1	802.5	653.4	0.42	0.31	0.34	0.12	1.23	0.75
502	8337.5	18629.1	3936.7	0.5	0	0	0	0	0	0	0	0	7726.0	0.0	0.0	0.00	0.15	0.00	0.15	#DIV/0!	#DIV/0!
504	8889.8	12438.1	5926.9	641.5	29822.7	51769.4	13768.6	82705.8	26429.8	12273.3	39080.1	24199.2	6974.1	44516.6	25495.6	6.38	0.10	3.66	0.08	1.75	0.38
506	1198	318.5	17.1	0	1528.8	2425.2	1443.3	10157.1	2476.9	6840.5	836.4	2955.9	383.4	3888.6	3277.4	10.14	0.22	8.55	0.11	1.19	0.82
803	432.6	1755.5	684.3	112.7	21.6	1577.8	69.4	107.8	117.7	86.4	68.3	20.8	746.3	444.2	73.3	0.60	0.11	0.10	0.15	6.06	0.40
1104	3065.1	5228.1	952.3	303.8	27.7	2404.8	1776	92.6	244.6	208.9	3.3	2599.8	2387.3	1075.3	764.2	0.45	0.26	0.32	0.37	1.41	0.79
1201	4114.4	1802.9	900.2	707.7	91.8	920.8	342.5	1534.1	915.7	278.7	87.8	1363.5	1881.3	722.3	661.4	0.38	0.34	0.35	0.22	1.09	0.86
1202	4009.5	2174.8	1789	1676.1	4932.1	4575.8	8618.8	2002.7	4703.7	2031.2	2491.4	6617	2412.4	5032.4	3960.8	2.09	0.17	1.64	0.27	1.27	0.67
1205	566.2	311.3	261.4	75.6	53.9	2100.5	494.9	20.9	310.8	365.4	16.1	187.7	303.6	667.6	220.0	2.20	0.52	0.72	0.45	3.03	0.40
1303	2107.9	371.1	98.3	71.9	310.7	646.4	113.4	1007.3	819.2	726.2	154.9	36.9	662.3	519.5	434.3	0.78	0.82	0.66	0.57	1.20	0.80
1304	2528.6	269.6	451.8	110.9	604.6	1505.6	477.6	1430	1438	10.5	79.1	28	840.2	1004.5	388.9	1.20	0.84	0.46	0.13	2.58	0.34
1306	1564.8	886.9	127.6	169.3	13.5	561.3	1255.7	128.9	55.6	9.4	49.9	65.3	687.2	489.9	45.1	0.71	0.74	0.07	0.16	10.87	0.22
1401	7892.1	4937.3	1212.1	2079.9	3194.6	3497.7	3639.5	4400.2	2689.1	2711.1	1347.6	3172.8	4030.4	3683.0	2480.2	0.91	0.85	0.62	0.35	1.48	0.05
1402	1860.9	1529.5	701	549.6	410.1	1193.2	970.2	49.4	314.7	222.2	56.4	121.2	1160.3	655.7	178.6	0.57	0.25	0.15	0.03	3.67	0.18
1404	173.8	672	121.6	3.1	3427.7	7172.5	1740.6	6597.2	2865.9	2396.5	3778.5	2106.1	242.6	4734.5	2786.8	19.51	0.04	11.49	0.01	1.70	0.32
1406	815.5	0	0	0	1133.1	1786.1	657.6	1499.2	1267.9	33.6	146.3	8.3	203.9	1269.0	364.0	6.22	0.05	1.79	0.21	3.49	0.13
1501	4278.5	1397.2	1443.7	941.4	621.3	2372.1	1721	4590.7	733	676	39.8	211.9	2015.2	2326.3	415.2	1.15	0.85	0.21	0.10	5.60	0.13
1502	2122.9	1646.5	1252	1510.8	2140	3529.7	2774.9	14690.2	6650.5	5982.4	1042	1474.5	1633.1	5783.7	3787.4	3.54	0.27	2.32	0.20	1.53	0.65
1504	2129.5	401.2	911.3	118.1	14.5	406.5	393.5	5230	152.5	1023.2	110.6	41.5	890.0	1511.1	332.0	1.70	0.72	0.37	0.39	4.55	0.45
1506	0	305.4	0	0	2578.7	1004.5	0	14417	1322.3	2523.7	326.6	3570.7	76.4	4500.1	1935.8	58.94	0.28	25.35	0.07	2.32	0.43
1601	1927.4	66.8	1216.3	249.5	472.9	1666.9	1567.1	2240.2	1161.7	2032.7	436.1	745	865.0	1486.8	1093.9	1.72	0.48	1.26	0.75	1.36	0.52
1602	1053.4	619.8	509.9	161.1	121.2	131	187.2	327.6	188.8	43.2	44.9	20.9	586.1	191.8	74.5	0.33	0.18	0.13	0.04	2.58	0.23
1603	1015.4	127.2	312.5	299.1	0	1221.6	818.8	656.4	327	308.4	216.7	0	438.6	674.2	213.0	1.54	0.63	0.49	0.31	3.16	0.19
1604	92.5	17.8	531.6	47.8	0	126	216.2	121.2	205.4	433.2	156.8	0	172.4	115.9	198.9	0.67	0.60	1.15	0.88	0.58	0.48
1605	2591.9	55.1	15.6	0	639.4	3511.9	407.9	2763.6	1604.9	310.5	654.9	137.9	665.7	1830.7	677.1	2.75	0.41	1.02	0.98	2.70	0.35
1802	6739.5	399.4	3216	1021.9	1844.7	989	3959.9	1806.6	3407.7	1113.7	791.8	636.3	2844.2	2150.1	1487.4	0.76	0.65	0.52	0.24	1.45	0.56
1804	669.5	2.6	643.1	276.9	716.8	456.2	936	281.7	521.5	329.1	161.2	260.3	398.0	597.7	318.0	1.50	0.16	0.80	0.66	1.88	0.20
1901	12758	9566.8	12544	8696.2	9560.6	19103.6	15327	7765.7	11132.6	20557.8	13891.4	6698.6	10891.3	12939.2	13070.1	1.19	0.52	1.20	0.52	0.99	0.88
2103	95.6	29.5	1285.9	1800.8	799.8	0	486.9	665.5	2506.8	0	24.7	995.1	803.0	488.1	881.7	0.61	0.50	1.10	0.93	0.55	0.46
2201	8937.4	3735.8	6264.4	4105.4	2995.6	3498.2	5574.3	1320	2748.6	4945.5	1916	3138.9	5760.8	3347.0	3187.3	0.58	0.16	0.55	0.22	1.05	0.91
2202	8393	5120.6	4825	2829.4	3115.3	4753.8	5088.4	4123.3	3230.4	7665.7	2279.8	8859.1	5292.0	4270.2	5508.8	0.81	0.53	1.04	0.94	0.78	0.51
2203	2720.4	1919.2	3562.8	3678.8	112.7	5607.5	3107.3	1681.9	78.3	0	185.6	3061.9	2970.3	2627.4	831.5	0.88	0.82	0.28	0.04	3.16	0.33
2204	11565	11445.3	3902.1	7342.8	1485.2	8289.2	5841.8	2228.7	1185.6	3091.8	2268.5	3826.5	8563.8	4461.2	2593.1	0.52	0.20	0.30	0.06	1.72	0.31
2205	76.3	1022.4	1194	859.9	648.1	2315.1	1087.7	927	618.1	0	36.6	156.1	788.2	1244.5	202.7	1.58	0.24	0.26	0.23	6.14	0.12
2207	412.5	368.6	773.3	572.9	256.2	872.5	1137.9	264.4	397.4	112.9	639.4	890.9	531.8	632.8	510.2	1.19	0.64	0.96	0.87	1.24	0.72
2208	721.5	2118.6	499.5	104.5	256	27.2	1063	50	295.8	653.9	5	415	861.0	349.1	342.4	0.41	0.43	0.40	0.25	1.02	0.99
2301	335.6	10.7	633.9	909	12.7	1130	624.9	349.4	303.5	100.3	119.1	547.1	472.3	529.3	267.5	1.12	0.89	0.57	0.24	1.98	0.46
2302	330.8	754.2	1471.4	666.4	221.4	751.2	1291.7	847.1	323.1	1490	49.2	234.6	805.7	777.9	524.2	0.97	0.75	0.65	0.58	1.48	0.60
2303	6374.6	8046.1	4764.5	6314.3	9656.6	18979	9174	4781.1	7438.8	10732.7	5784.2	10025	6374.9	10647.7	8495.2	1.67	0.19	1.33	0.05	1.25	0.50
2304	44.2	91.9	70.7	41.3	385.3	1715.4	626.9	32.7	703.5	177	60.2	494.6	62.0	690.1	358.8	11.13	0.17	5.79	0.15	1.92	0.53
2401	721.8	2032.7	1192.1	417.2	549.6	669.8	1732.2	113.7	43.6	1082.1	481.2	1668.7	1091.0	766.3	818.9	0.70	0.47	0.75	0.63	0.94	0.94

2402	3142.4	11548.8	4275.5	3882.4	3456.4	7185.5	5197.7	3274.8	1985.5	16338	9361.5	3631.8	5712.3	4778.6	7829.2	0.84	0.49	1.37	0.29	0.61	0.28
2404	5502.8	1703.4	4829.9	5311.6	5021.1	1835.4	4023.6	2000.8	3104.4	2716.1	2188.5	3810.7	4336.9	3220.2	2954.9	0.74	0.24	0.68	0.20	1.09	0.80
2405	378	717.3	773.8	30.2	87.2	1123.6	158.9	216.6	153.9	440.4	5581.9	74.1	474.8	396.6	1562.6	0.84	0.76	3.29	0.45	0.25	0.47
2406	7375.4	9936.9	6610.9	5904.5	4568.7	8226.9	4569.6	2134.3	4376.5	8470	5245.4	5884.4	7456.9	4874.9	5994.1	0.65	0.01	0.80	0.10	0.81	0.30
2407	114.3	223	2262.5	904.2	1015.8	1201.7	2378.8	908.3	771.6	956.6	1742.9	2352.4	876.0	1376.2	1455.9	1.57	0.15	1.66	0.25	0.95	0.87
2408	1820.6	1037	373.5	1477.4	1436.1	2745.4	992.6	1774.2	634.3	2782.6	290.8	6097.2	1177.1	1737.1	2451.2	1.48	0.29	2.08	0.39	0.71	0.60
2409	1474.2	133.5	693.6	79.2	990.2	953	900.2	612.8	789	681.9	309	402.4	595.1	864.1	545.6	1.45	0.41	0.92	0.88	1.58	0.04
2501	929.3	140.5	4589.1	3783.9	515.3	418.2	571.3	1097.5	1250.9	683.9	12531.2	366.2	2360.7	650.6	3708.1	0.28	0.18	1.57	0.61	0.18	0.38
2502	6727.2	2331.3	3156.1	3209	0	0	10272.6	0	1990.9	606	9947.8	0	3855.9	2568.2	3136.2	0.67	0.69	0.81	0.80	0.82	0.35
2503	3126.2	542.3	2108.1	1490.8	1907.9	1416.5	2770.7	2295.9	1455.1	442.5	1051.2	703.3	1816.9	2097.8	913.0	1.15	0.61	0.50	0.07	2.30	0.03
2505	1622	69	1173.4	1670.2	548.9	3035.1	2469.2	1342.4	1193.7	2046.3	432.6	1828.9	1133.7	1848.9	1375.4	1.63	0.48	1.21	0.72	1.34	0.51
2506	123.7	65	859.8	581.3	75.9	640.3	1147.3	375.5	209.1	527.8	543.9	890.3	407.5	559.8	542.8	1.37	0.45	1.33	0.48	1.03	0.95
2602	1720.1	1126.5	3202.4	1609.6	231	1716.7	2450.2	1019.9	742.2	1889.3	723.2	278.9	1914.7	1354.5	908.4	0.71	0.28	0.47	0.23	1.49	0.44
2603	667.8	39.2	104.2	818.1	0	354.5	8549	581.5	386.5	331	152.2	23.4	407.3	2371.3	223.3	5.82	0.43	0.55	0.49	10.62	0.38
2608	1139.4	541.4	3990.5	623.5	74.7	1277	597.6	412.4	1670.6	503.1	502.5	68.2	1573.7	590.4	686.1	0.38	0.35	0.44	0.39	0.86	0.87
2609	2504.4	74.5	2310.9	1140.8	78.9	311.2	1424.1	0	1021.4	339.9	606.6	128.4	1507.7	453.6	524.1	0.30	0.15	0.35	0.11	0.87	0.86
2610	740.4	0	170.4	801.6	380.2	1489.7	1022.7	999.1	164.7	0	109.2	0	428.1	972.9	68.5	2.27	0.27	0.16	0.16	14.21	0.04
2611	1521.3	540	612.9	1947.8	0	884.4	713.2	19.5	105.4	3258.4	19.1	49.4	1155.5	404.3	858.1	0.35	0.28	0.74	0.79	0.47	0.54
2613	94.5	556.5	22.4	233.1	85.6	2401	1895.8	1270.3	138.9	0.9	55	695.6	226.6	1413.2	222.6	6.24	0.08	0.98	0.99	6.35	0.13
2614	1038.6	1.4	938.7	1921.8	34.7	222	430.8	265.5	128.1	832	68.3	254.5	975.1	238.3	320.7	0.24	0.16	0.33	0.30	0.74	0.71
2801	975.3	0	784	468.8	505.6	383.2	1144.9	299.8	474.1	0	110	111.4	557.0	583.4	173.9	1.05	0.91	0.31	0.08	3.36	0.16
2902	1264.7	63	806.5	4.8	674.1	259.2	1162.5	382.5	345.8	11.1	583.7	78.4	534.8	619.6	254.8	1.16	0.74	0.48	0.30	2.43	0.02
2904	862.3	846.4	877.4	242.2	713.8	284.6	240.3	742.6	2300.8	95.2	1501.2	1456.1	707.1	495.3	1338.3	0.70	0.48	1.89	0.29	0.37	0.12
3201	15498.2	29153.6	5219	10972.1	3803.3	14022.5	13418.5	11889	4841.5	2662.2	9443.1	10427.8	15210.7	10783.3	6843.7	0.71	0.48	0.45	0.31	1.58	0.24
3203	2260.6	4829.8	374.4	5	684.2	118.7	574.8	474.2	759.7	8.3	12.6	495.6	1867.5	463.0	319.1	0.25	0.32	0.17	0.28	1.45	0.39
3204	103.3	34.3	51	45.7	16.3	9.4	288.5	112.1	34.2	0	6.7	145.9	58.6	106.6	46.7	1.82	0.55	0.80	0.78	2.28	0.48
3205	1647	7005.1	1682.3	2702.4	345.8	1791.4	2008.1	1246.8	1988.4	840.7	1578.2	11482.2	3259.2	1348.0	3972.4	0.41	0.20	1.22	0.83	0.34	0.39
3206	169.8	1646.1	122.2	493.9	0	24	725.9	344.5	325.9	0	0	553.1	608.0	273.6	219.8	0.45	0.52	0.36	0.43	1.25	0.83
3301	9658.4	25069.1	6284.2	10141.1	7449.2	15229.7	7153.2	4751.7	5760.6	11087.3	5925.5	10533	12788.2	8646.0	8326.6	0.68	0.17	0.65	0.27	1.04	0.89
3302	2515.6	2116.1	5032.5	3594.5	2324.7	4013.2	3346.3	468.1	7851.5	1865.4	2776.7	1935	3314.7	2538.1	3607.2	0.77	0.52	1.09	0.88	0.70	0.57
3303	3243.1	4534	3773.1	2955.9	571.5	4554.8	2691.2	1374.9	1810.2	2279.6	1852.4	2351.8	3626.5	2298.1	2073.5	0.63	0.10	0.57	0.02	1.11	0.80
3402	10179.7	18853.9	5417.4	9600.3	6379	9103	7537.9	5173.4	6731.8	7898.3	6470.7	9329.4	11012.8	7048.3	7607.6	0.64	0.20	0.69	0.29	0.93	0.68
3501	2882.6	500.5	1969.7	905.8	189	2150.5	1300.6	651.4	868	456.2	808.3	1298.6	1564.7	1072.9	857.8	0.69	0.62	0.55	0.29	1.25	0.73
3503	1447.9	188.8	3213	2254.4	1671.5	1419.3	2193.9	1167.3	2119.9	2049.9	9552.8	2124.7	1776.0	1613.0	3961.8	0.91	0.79	2.23	0.23	0.41	0.26
3504	2046.2	633.1	787.7	1371.5	905.5	299.8	2311.6	872.1	524.1	374.3	705	2189.9	1209.6	1097.3	948.3	0.91	0.86	0.78	0.63	1.16	0.82
3506	12503.6	4324	6782.1	6570.1	4944.8	3415.7	6533.4	3520	5180	16054.6	4203.5	7297.7	7545.0	4603.5	8184.0	0.61	0.17	1.08	0.88	0.56	0.35
3507	1231.5	280.9	194	91.1	25.7	154.7	123.8	193.3	299.6	1295.9	258.2	99.8	449.4	124.4	488.4	0.28	0.35	1.09	0.93	0.25	0.27
3508	2633.2	76.9	1009.1	1229	678.8	3199.8	1738.8	1054.1	1080.4	1037.8	1149.3	1990.3	1237.1	1667.9	1314.5	1.35	0.71	1.06	0.90	1.27	0.64
3509	110.5	31.2	8.2	48.1	113.6	152.9	200	44.2	742.6	15.7	887.7	342.1	49.5	127.7	497.0	2.58	0.20	10.04	0.11	0.26	0.15
3601	1284.4	1932.2	993.7	1318.8	831.1	2533.9	1193.1	695.3	960.6	2092	1399	2195.4	1382.3	1313.4	1661.8	0.95	0.82	1.20	0.35	0.79	0.46
3602	131.5	34.6	924	989.2	21.8	832	620.8	10.3	452.1	936.6	464	0	519.8	371.2	463.2	0.71	0.71	0.89	0.90	0.80	0.51
3605	6421.4	11461.2	4280	5629.3	5964	10995.8	5284.4	3434.9	3989.5	8308	5326.6	8387.6	6948.0	6419.8	6502.9	0.92	0.48	0.94	0.77	0.99	0.96
3606	1774.9	1452.9	1068.2	1401.6	788	1711.9	914.5	544	839.3	1219.2	748.5	1062.4	1424.4	989.6	967.4	0.69	0.24	0.68	0.07	1.02	0.92
3607	654.4	556.6	544.1	432.7	376.9	661.2	112	311.3	285.7	368.4	497.6	469.3	547.0	365.4	405.3	0.67	0.21	0.74	0.21	0.90	0.80
3609	1138.3	312.8	223.7	652.4	95.2	680.2	1340.7	493.1	782.4	98	472.7	752.8	581.8	652.3	526.5	1.12	0.89	0.90	0.72	1.24	0.75
3610	1996.2	153.7	780.5	721.2	71.3	7.3	2082.5	1681.5	382.5	1350.8	21	649.4	912.9	960.7	600.9	1.05	0.95	0.66	0.64	1.60	0.66
3611	3919.6	5959.1	1710.8	2133.3	355	3573.5	1943	2140.4	1713.3	3972.3	1927.5	2128.5	3430.7	2003.0	2435.4	0.58	0.22	0.71	0.22	0.82	0.27
3703	5889	13454.6	2426.6	2234.8	1743.4	3268.4	9016	5431.6	6715.3	5880.7	6222.3	9036.3	6001.3	4864.9	6963.7	0.81	0.78	1.16	0.78	0.70	0.31
3704	3334	7085.8	6082.9	7643.7	7284.1	9194.5	5201.8	1927.5	2511.1	3940.6	3099	8885.3	6036.6	5902.0	4609.0	0.98	0.95	0.76	0.26	1.28	0.68
3705	1213.6	677.4	4710.6	4547.3	2455.9	3281.3	3984.5	2083.1	1799.8	2943.9	3311.8	2013.8	2787.2	2951.2	2517.3	1.06	0.89	0.90	0.82	1.17	0.06
3706	3957.8	220.2	2283.8	717.9	130.9	795.4	5731.9	1732.2	2415.8	2243.4	3233	6041.7	1794.9	2097.6	3483.5	1.17	0.85	1.94	0.32	0.60	0.40
3801	2920.1	375.5	1924.6	3376	1667.1	2337.4	4209.7	2931.6	908	666.3	1176.3	2500.4	2149.1	2786.5	1312.8	1.30	0.52	0.61	0.17	2.12	0.09
3805	366.8	1797.5	693.1	1217.7	919.3	1353.1	2012.7	1276.7	0	79.8	256	783.9	1018.8	1390.5	279.9	1.36	0.40	0.27	0.11	4.97	0.03

3807	6165.9	2404.4	6458.7	8133.1	3454.3	3327.6	6726.4	2166.4	5186.4	5119.7	3448.2	4982.7	5790.5	3918.7	4684.3	0.68	0.32	0.81	0.48	0.84	0.62
3809	2300.4	882.5	1452.1	4365.6	3795.5	3855.1	6183	3560.6	2576.5	1028.4	868.4	5025	2250.2	4348.6	2374.6	1.93	0.17	1.06	0.66	1.83	0.26
4204	210.2	2178	1.7	0	280.9	0	108.2	864.4	140.8	251.3	25.8	149.9	597.5	313.4	142.0	0.52	0.69	0.24	0.42	2.21	0.46
4303	12.8	4.9	1186.7	284.3	322.3	229.7	268.5	481.9	215.2	212.1	209.1	442.6	372.2	325.6	269.8	0.87	0.88	0.72	0.75	1.21	0.06
4305	3218.1	682.1	3192	2262.7	1955.7	767.9	1039.7	931	1495.8	3261.6	280.8	425.1	2338.7	1173.6	1365.8	0.50	0.09	0.58	0.48	0.86	0.82
4307	1372.8	0	151.8	393.5	0	0	0	0	53.7	15.3	22.6	65	479.5	0.0	39.2	0.00	0.22	0.08	0.24	0.00	0.05
4308	141.6	242.1	57	874	102.4	737.4	518.8	246.5	733.4	581.1	698	103.3	328.7	401.3	529.0	1.22	0.80	1.61	0.59	0.76	0.54
4309	371.3	19.3	11	277.7	0	160.8	711.2	824.1	705.6	314.9	215.5	112.5	169.8	424.0	337.1	2.50	0.37	1.99	0.24	1.26	0.80
4402	3426.8	5195.6	2091	3286.4	1216.4	2147.2	2823.3	1671	2976.1	2895.7	1759.6	3663.7	3500.0	1964.5	2823.8	0.56	0.15	0.81	0.32	0.70	0.30
4405	1238.9	2264.4	1350	2228.4	1272.5	1754.9	3122.1	2270.4	2076.8	5031.3	2123.2	3894.6	1770.4	2105.0	3281.5	1.19	0.55	1.85	0.05	0.64	0.28
4406	258.8	905.2	148.3	799.1	207.8	28.9	63.9	24.6	284.4	22.4	21.6	69.5	527.9	81.3	99.5	0.15	0.14	0.19	0.15	0.82	0.54
4408	838.6	123.8	0	0	237.2	1092.4	1493.4	637.9	285.2	529.7	0	0	240.6	865.2	203.7	3.60	0.26	0.85	0.86	4.25	0.13
4501	4831.1	1899.5	3012.5	3928.2	1256.5	0	2897.8	2206	3374.4	213.7	2325.1	4677.5	3417.8	1590.1	2647.7	0.47	0.08	0.77	0.26	0.60	0.25
4503	353.1	0	0	909.6	0	8084	1015.4	0	975.4	397.8	527.5	654.9	315.7	2274.9	638.9	7.21	0.42	2.02	0.20	3.56	0.48
4504	1469.1	0	0	445.8	0	1239.2	1510.4	0	1136.6	8808.1	222.3	183.6	478.7	687.4	2587.7	1.44	0.79	5.41	0.42	0.27	0.40
4601	642.2	3808.2	956.2	1154.5	543.7	201	1350.3	1511.2	1883	3859	1057.5	2292.9	1640.3	901.6	2273.1	0.55	0.50	1.39	0.14	0.40	0.20
4602	851.7	3587.3	877.5	786.7	0	0	1227.2	494.3	1389.2	1549.5	1071.5	3161.4	1525.8	430.4	1792.9	0.28	0.30	1.18	0.79	0.24	0.10
4603	142.2	2153.1	1764.6	3352.6	2395.6	5563.8	1617.3	221.3	3832.9	34.1	3175.1	1603.4	1853.1	2449.5	2161.4	1.32	0.71	1.17	0.84	1.13	0.88
4604	3338.2	1986.1	45.1	1499.1	699.1	1029.3	831.4	1370.8	511.2	1994.1	57.9	1292.8	1717.1	982.7	964.0	0.57	0.39	0.56	0.36	1.02	0.96
4801	6273.1	2373.1	1398.3	1857.5	2121.6	3659.1	3011.5	4659.5	1954.6	5439	1842	2560.6	2975.5	3362.9	2949.1	1.13	0.82	0.99	0.99	1.14	0.65
4901	3604.1	1594.2	2849.8	4713.4	4353.8	360.6	5096.2	2303.1	1744	325	3341.4	5860.8	3190.4	3028.4	2817.8	0.95	0.89	0.88	0.64	1.07	0.89
4902	3088	549.6	1326.2	992.4	1532.7	228	2686.8	2337.3	952.7	1048.3	1080.6	3264.6	1489.1	1696.2	1586.6	1.14	0.79	1.07	0.92	1.07	0.87
4906	8668.8	3247	588.8	562.4	0	2933.3	1628.6	3304.4	2594.7	5053.6	1878.6	1835	3266.8	1966.6	2840.5	0.60	0.64	0.87	0.84	0.69	0.42
5202	447.8	141.4	57.1	346.7	35.8	465.2	131.1	134.7	86.4	0	656	356.3	248.3	191.7	274.7	0.77	0.75	1.11	0.91	0.70	0.72
5203	2361.7	349.2	37.8	473.5	4692.3	13608.7	2546.5	20031.8	5052	5927.4	519.2	3603.9	805.6	10219.8	3775.6	12.69	0.11	4.69	0.07	2.71	0.18
5306	3344.2	2081.2	104.1	316.4	65.4	3777	429.3	561.1	233.8	1155.6	340.8	484.4	1461.5	1208.2	553.7	0.83	0.83	0.38	0.33	2.18	0.39
5307	718.6	0	70.4	1688.9	0	737.8	144.9	2124.6	1848.5	4811	159.6	295.1	619.5	751.8	1778.6	1.21	0.70	2.87	0.45	0.42	0.48
5412	1101.9	734.8	2827.8	227.4	266.7	2571.3	328.9	15.7	1017.6	672.3	1392.4	1347.6	1223.0	795.7	1107.5	0.65	0.67	0.91	0.84	0.72	0.70
5414	2523.3	173.7	220.3	662	1102.8	2860.8	1038.7	1102.5	1197.6	1127.5	1046.5	497.5	894.8	1526.2	967.3	1.71	0.51	1.08	0.90	1.58	0.28
5503	9428.7	3564.4	1579.5	3721.8	3288.3	6835.3	5159	6591.6	3073.3	5380.6	3988	4979.9	4573.6	5468.6	4355.5	1.20	0.73	0.95	0.92	1.26	0.04
5507	1603.1	559.2	192.2	357	453	2482.6	1726.4	2186.2	946.9	704.1	208.7	1404.9	677.9	1712.1	816.2	2.53	0.25	1.20	0.72	2.10	0.18
5508	44.2	6	51.1	309.8	977.3	38.3	640.1	980	1285.6	4262.9	669.1	52.1	102.8	658.9	1567.4	6.41	0.06	15.25	0.23	0.42	0.48
5601	3464	1917.4	1228.1	2165.4	3706.2	1807.8	2587.1	4304.2	2121.5	10184.4	2631.7	2161.6	2193.7	3101.3	4274.8	1.41	0.18	1.95	0.40	0.73	0.66
5602	244.2	40.1	196.4	1.5	90.1	486.7	489.8	364.6	47.4	20.9	4.3	93.1	120.6	357.8	41.4	2.97	0.18	0.34	0.34	8.64	0.05
5604	1013.7	331.6	325.2	84.2	536.8	693.3	509.4	530.3	289.1	149.5	621.8	584.9	438.7	567.5	411.3	1.29	0.58	0.94	0.93	1.38	0.38
5605	3497.9	2810.4	3506.3	6873.7	3337.9	4287.1	600.2	4425.6	1944.6	6635.2	2777.4	542.6	4172.1	3162.7	2975.0	0.76	0.40	0.71	0.61	1.06	0.91
5606	5142.5	2824	2326.4	7938	7174.2	7286.8	5202.7	6435.9	6135.4	7081.9	4620.3	6164.5	4557.7	6524.9	6000.5	1.43	0.22	1.32	0.34	1.09	0.07
5607	5752.7	1142.5	0	0	0	8153.6	5306.1	0	2498.7	4703.9	4638.6	2799.8	1723.8	3364.9	3660.3	1.95	0.61	2.12	0.35	0.92	0.85
5608	396.7	0	381.5	48.8	174.5	426.7	406.3	353.1	346.6	460.4	151	1190.1	206.8	340.2	537.0	1.65	0.43	2.60	0.36	0.63	0.46
5706	148593.9	145273.7	149765.3	162504.4	131335.8	122113.3	139253.5	59784.9	106393.6	124562	144507.2	93565.2	151534.3	113121.9	117257.0	0.75	0.17	0.77	0.09	0.96	0.75
6110	119.9	18.3	1389.9	1059.5	993.2	2452.8	1069.9	331.5	1391.3	355.1	809.3	936.5	646.9	1211.9	873.1	1.87	0.48	1.35	0.61	1.39	0.62
6202	4230.7	1545.5	1557.6	87.4	626.5	6044.1	1754.4	1865.7	2185.9	5428.5	10159.2	1374.2	1855.3	2572.7	4787.0	1.39	0.70	2.58	0.28	0.54	0.37
6204	1550.3	35.3	25.5	0	42.1	6.4	16.8	0.8	63.3	0	3.7	35.8	402.8	16.5	25.7	0.04	0.38	0.06	0.38	0.64	0.48
6306	367.3	899.5	906.2	446.8	110.5	1118.9	413.4	226.4	669.1	90	683.8	419.9	655.0	467.3	465.7	0.71	0.30	0.71	0.48	1.00	1.00
6307	155.1	0	159.6	0	83.7	0	322.7	1000	383.9	49	1755	405.4	78.7	351.6	648.3	4.47	0.35	8.24	0.20	0.54	0.53
6402	603.9	565.1	1276.4	339.6	377.6	540.4	849	2276	817	444.4	498.4	430.7	696.3	1010.8	547.6	1.45	0.61	0.79	0.55	1.85	0.41
6502	888.7	534.7	828	718.5	271	635	1099.5	328.5	1098.9	228.2	672.9	640.5	742.5	583.5	660.1	0.79	0.50	0.89	0.50	0.88	0.82
6503	1632.3	1120.7	39.1	659	723	2690.8	989	1760.4	1664.7	1119	1408.5	502.8	862.8	1540.8	1173.8	1.79	0.30	1.36	0.45	1.31	0.59
6504	319.9	15.6	588.4	230.3	162.8	829.4	1023.4	94.9	916.9	346.1	478.3	326.3	288.6	527.6	516.9	1.83	0.38	1.79	0.23	1.02	0.97
6601	9877.2	18871.2	14171.7	12513.6	8621.1	13712	15135.2	6414.3	11715.2	6051	14506.1	4171.1	13858.4	10970.7	9110.9	0.79	0.18	0.66	0.27	1.20	0.47
6602	2858.9	68.3	1327.9	2105.7	1565.7	1981.2	2463.4	1639.3	1822	1233.3	2265.5	1355.2	1590.2	1912.4	1669.0	1.20	0.69	1.05	0.90	1.15	0.32
6703	183118.7	207505.8	193407.8	232396.3	160553.9	95491.9	130718.5	42461.8	140315.5	140840	160631.6	177660.2	204107.2	107306.5	154861.9	0.53	0.07	0.76	0.01	0.69	0.24
7202	2157.5	7068.1	2438	6565.3	1772.6	8929.4	5970.1	10324.3	25618.7	45307.8	24253.9	16209.2	4557.2	6749.1	27847.4	1.48	0.11	6.11	0.03	0.24	0.04

7204	3674.6	3185.2	3539.3	571.1	30388.4	12973.8	2704.4	5618.2	6277.6	9192.1	4851.2	5706.6	2742.6	12921.2	6506.9	4.71	0.18	2.37	0.04	1.99	0.37
7206	2963.4	2783.6	1496.3	1460.1	5957.1	50515.2	880.4	6479.4	4243.2	2449.1	2384	7105.1	2175.9	15958.0	4045.4	7.33	0.31	1.86	0.25	3.94	0.40
7207	4706.9	5688.5	1097.6	2545.8	2862.4	2433	3841.8	4686.7	3696.4	794.7	1943.3	4251.3	3509.7	3456.0	2671.4	0.98	0.97	0.76	0.61	1.29	0.30
7208	2549.4	652.1	171.9	836.6	2131.6	66.6	1976.5	3232.2	3321.3	42.4	602.8	1161.1	1052.5	1851.7	1281.9	1.76	0.37	1.22	0.49	1.44	0.49
7209	161.1	1485.9	0	0	16	785.7	202.3	126	341.7	81.4	344.1	96.5	411.8	282.5	215.9	0.69	0.57	0.52	0.66	1.31	0.79
7302	12735.2	11507.6	13218.2	7860.9	6802.2	8068.5	4449.1	1531.7	6330.3	6323.5	6690.6	5858.4	11330.5	5212.9	6300.7	0.46	0.01	0.56	0.02	0.83	0.48
7303	141.2	251.3	933.8	464.5	3756	5344.1	627	5184.5	1889.7	852.4	478.9	2125	447.7	3727.9	1336.5	8.33	0.08	2.99	0.18	2.79	0.08
7304	386.1	2684.4	605.5	882.7	1321	3783.5	372.2	985.2	1343.8	256.3	9801.1	518.3	1139.7	1615.5	2979.9	1.42	0.24	2.61	0.52	0.54	0.66
7401	239.5	0	81.5	488.2	25.3	208.3	1461.5	1184.7	363.8	39.7	17.3	158.7	202.3	720.0	144.9	3.56	0.23	0.72	0.60	4.97	0.25
7402	0	121.4	69.7	481.5	11337.1	79.5	514.9	0	1644.3	204	51.1	799	168.2	2982.9	674.6	17.74	0.40	4.01	0.28	4.42	0.42
7403	1295.7	349.2	56.9	0	2327.1	27	394.7	299.9	275.3	60.7	159.1	533.3	425.5	762.2	257.1	1.79	0.31	0.60	0.65	2.96	0.41
7404	1972.3	2107.4	114.3	430.8	7112	16154.9	3618.9	13554.1	5985.8	3521.1	6795.5	8316.5	1156.2	10110.0	6154.7	8.74	0.05	5.32	0.04	1.64	0.32
7405	771.6	239.9	974.1	795.3	487	2167.1	1221.2	905.9	201.8	331.1	634.4	163.1	695.2	1195.3	332.6	1.72	0.38	0.48	0.11	3.59	0.08
7406	53	46.2	54.4	137.5	419.8	1688.2	86.5	20.2	771.4	101.4	539.1	1.6	72.8	553.7	353.4	7.61	0.32	4.86	0.25	1.57	0.70
7503	26.8	38.1	43.1	13.7	1190.9	89.1	215.3	178.8	287.6	15.4	45.1	78.2	30.4	418.5	106.6	13.76	0.23	3.50	0.32	3.93	0.21
7504	54.7	66.7	112.2	337.7	72.5	42.6	635.1	295.8	587.2	285.1	141.7	519.8	142.8	261.5	383.5	1.83	0.45	2.68	0.11	0.68	0.61
7601	5823.9	7093.8	3427.5	1784.7	2323.2	4183.9	2861.7	1192.4	3486.8	1273.9	2431.8	2977.1	4532.5	2640.3	2542.4	0.58	0.09	0.56	0.27	1.04	0.93
7602	722	31.5	450	591.2	110.1	407.6	658.5	517.1	740.6	157.8	382.7	461.7	448.7	423.3	435.7	0.94	0.91	0.97	0.83	0.97	0.96
7604	1486.9	0	1126.9	1824.3	698	939	1372.7	125.7	917.9	334.2	817.1	0	1109.5	783.9	517.3	0.71	0.61	0.47	0.28	1.52	0.26
7605	1448.1	594.2	1685.5	1548.4	1830.9	1877.5	3108.9	1533.2	1540.2	12.9	759.1	1004.4	1319.1	2087.6	829.2	1.58	0.11	0.63	0.10	2.52	0.09
7705	91856.2	125991.5	120898.4	120376.2	105763	59002	77794.2	10436.4	106228.9	93493.2	79436.6	112486.7	114780.6	63248.9	97911.4	0.55	0.14	0.85	0.27	0.65	0.24
7706	4844.5	3068.2	2704.5	2864.4	4557	3261.4	5785.5	2233.8	3899.9	896.4	10554.9	8454.6	3370.4	3959.4	5951.5	1.17	0.54	1.77	0.37	0.67	0.41
8203	4766.4	5196.1	1841.1	630.9	584.5	12551.9	9183.8	60364	1898.8	31229	0	29422.5	3108.6	20671.1	15637.6	6.65	0.31	5.03	0.24	1.32	0.66
8205	2630.5	2745.6	294.3	1415.4	3041.6	4084.7	9216.6	4918.7	2049.3	0	3443.9	4987.9	1771.5	5315.4	2620.3	3.00	0.16	1.48	0.62	2.03	0.14
8206	31.6	6951.6	138.8	162.7	16366.8	131	30.9	5764	2126.2	2343.8	18791.6	18.6	1821.2	5573.2	5820.1	3.06	0.50	3.20	0.49	0.96	0.97
8302	1444.9	3015.9	2710.4	1198.3	204.8	3107.4	4003.8	1411.2	1523.4	287.6	416.8	1314.7	2092.4	2181.8	885.6	1.04	0.87	0.42	0.21	2.46	0.34
8303	1.2	100.1	380.9	117.9	186.6	105.6	59.6	6962.3	392.1	26.9	45.6	203.3	150.0	1828.5	167.0	12.19	0.40	1.11	0.92	10.95	0.40
8304	4.6	39.5	503.9	96	26	8.7	957	62.2	111.5	0	135.8	383.2	161.0	263.5	157.6	1.64	0.45	0.98	0.98	1.67	0.70
8401	7812.3	13539.4	2443.9	5332	25087.9	47019.4	18199.2	30798.7	22705.6	10775	23645.5	31177.9	7281.9	30276.3	22076.0	4.16	0.01	3.03	0.10	1.37	0.45
8403	811	367.7	640.5	66.7	522	948.5	205.3	138.6	1876.3	6.5	659.5	255.2	471.5	453.6	699.4	0.96	0.94	1.48	0.51	0.65	0.64
8404	145.6	17.3	33.8	471.4	24	1009.2	76.7	15.6	46.1	1227.3	15.2	233	167.0	281.4	380.4	1.68	0.74	2.28	0.57	0.74	0.26
8406	38.6	254.8	104	304.3	53	2476.8	1524.2	860.5	1227.5	93.2	8.2	524.3	175.4	1228.6	463.3	7.00	0.12	2.64	0.42	2.65	0.39
8502	344.3	19.1	354.5	207.8	17.3	568.7	174.6	86.1	679.9	0	33.8	152.4	231.4	211.7	216.5	0.91	0.93	0.94	0.92	0.98	0.99
8503	82.4	3331.7	95.2	2	163.8	10.4	57.5	149.8	422.3	0	11.9	2863.6	877.8	95.4	824.5	0.11	0.42	0.94	0.97	0.12	0.35
8601	1227.7	1272	4166.6	3310.2	2977.6	787.1	4758.2	1103	3489	112	1877.4	3498.7	2494.1	2406.5	2244.3	0.96	0.92	0.90	0.81	1.07	0.89
8603	2588.1	3179.5	3108.2	3928.9	3788.3	2032.4	8050.4	1215	7052.2	211.5	3304.6	5176.7	3201.2	3771.5	3936.3	1.18	0.75	1.23	0.66	0.96	0.94
8604	1105.4	1017.6	702.1	635.6	63	415.4	1221.7	214.4	1781.8	0	259.9	327.7	865.2	478.6	592.4	0.55	0.32	0.68	0.49	0.81	0.86
8605	227.6	1953	1059.7	457.9	0	60.6	2416.2	561	1456.3	0	454.1	0	924.6	759.5	477.6	0.82	0.82	0.52	0.54	1.59	0.72
8608	82.5	0	0	0	881.7	127.2	1591.1	237	90.1	0	0	627.7	20.6	709.3	179.5	34.39	0.13	8.70	0.38	3.95	0.30
8705	931.5	479.6	2508	718.7	0	586.5	3524.1	194.7	141.3	0	0	0	1159.5	1076.3	35.3	0.93	0.86	0.03	0.09	30.47	0.30
8710	3621.5	3407.4	3067.4	3041.8	0	1274.9	6025.9	601.9	669.8	0	1071.3	869.8	3284.5	1975.7	652.7	0.60	0.44	0.20	0.00	3.03	0.38
8711	2377	2457.6	3740.9	2507.5	328.7	1514.8	4824.5	1592.8	2045.3	664.2	1156.7	1833.1	2770.8	2065.2	1424.8	0.75	0.36	0.51	0.08	1.45	0.61
8901	133.7	0	44	995.9	487.3	129.9	554.4	45.2	761.9	0	92.3	127.7	293.4	304.2	245.5	1.04	0.98	0.84	0.89	1.24	0.73
9204	0	0	0	206.2	4741.3	1755.7	867.7	5239.9	3363.9	0	0	0	51.6	3151.2	841.0	61.13	0.06	16.31	0.43	3.75	0.10
9303	6253.9	0	88.5	0.6	161.9	66.4	290.8	7.2	215.2	0	86.8	128.4	1585.8	131.6	107.6	0.08	0.42	0.07	0.40	1.22	0.76
9304	116.2	0	13.1	138	558.3	1762.6	2672.8	965.3	94.7	0	288.5	459.2	66.8	1489.8	210.6	22.29	0.06	3.15	0.21	7.07	0.07
9403	0	0	0	0	4302.2	3849.2	2261.1	6844.4	562.6	29.9	1530.9	0	0.0	4314.2	530.9	#DIV/0!	0.02	#DIV/0!	0.23	8.13	0.06
9501	1679.7	1225.6	1846.1	388.5	626	1433.7	1489.3	171.6	851.2	1011	362.2	311	1285.0	930.2	633.9	0.72	0.27	0.49	0.14	1.47	0.41
9602	1673.2	1338.6	1899	47.2	332.4	425.3	474.3	52	1147.6	34.2	51.2	11.1	1239.5	321.0	311.0	0.26	0.07	0.25	0.10	1.03	0.97
9605	449.1	180.7	486.9	11.5	401.3	224.2	1426.6	144.6	1.5	66.8	0	282.05	282.1	549.2	87.6	1.95	0.32	0.31	0.35	6.27	0.27
