# Supplementary material for: Protein profile of Beta vulgaris leaf apoplastic fluid and changes induced by Fe deficiency and Fe resupply
Source: Front Plant Sci. 2015 Mar 18;6:145. doi: 10.3389/fpls.2015.00145 (PMC4364163; doi:10.3389/fpls.2015.00145)
Supplement: Supplementary file 5 [file Table4.PDF]

Table S4- Proteins identified in 2-DE IEF-SDS PAGE gels of sugar beet leaf apoplast. (a) spot number as in Figure 1S; (b) entry of protein hit using Mascot (codes in bold, underlined, and plain are entries in NCBI , EST, and sugar beet databases, respectively); (c) Plant species of the Mascot protein hit; (d) protein description; (e) e-blast values from blast searches of sugar beet and EST database entries; (f) UniProt entries of NCBI and blast hits; (g) Mascot score, number of peptides and ions (homology identification was retained with a probability of 95% and at least 2 matched peptides); (h) sequence coverage (a threshold of 10% was set for positive protein identification); (i) theoretical molecular weight and pI; (j) experimental molecular weight and pI; (k) GO:P description; (l) GO:P identifier.

SSP[a]	NCBI/Bv/EST entry[b]	Specie[c]	Protein description[d]	E-Blast[e]	UniProt[f]	Score/pep/ion[g]	Sec.cov %[h]	Mw/pI th[i]	Mw/pI exp[j]	Go:P[k]	GO:id[l]
**Carbon metabolism (53 spots, 27 Uniprot entries)**											
Glycolysis											
3703	CV301357	*Beta vulgaris*	similar to enolase (*Mesembryanthemum crystallinum*)	0.0	Q43130	993/21/13	59	29.9/5.4	96.7/5.7	glycolytic process	GO:0006096
3704	UMSBv_S13748_84980.t1	*Beta vulgaris*	similar to enolase (*Mesembryanthemum crystallinum*)	0.0	Q43130	1052/26/18	54	48.3/5.3	69.2/5.8	glycolytic process	GO:0006096
3705	UMSBv_S13748_84980.t1	*Beta vulgaris*	similar to enolase (*Mesembryanthemum crystallinum*)	0.0	Q43130	701/18/14	45	48.3/5.3	68.9/5.6	glycolytic process	GO:0006096
3706	UMSBv_S13748_84980.t1	*Beta vulgaris*	similar to enolase (*Mesembryanthemum crystallinum*)	0.0	Q43130	718/21/17	43	48.3/5.3	68.5/5.5	glycolytic process	GO:0006096
3801	UMSBv_S02502_165000.t1	*Beta vulgaris*	similar to 2,3-bisphosphoglycerate-independent phosphoglycerate mut	0.0	Q42908	740/23/19	50	61.5/5.3	85.7/5.5	glycolytic process	GO:0006096
2611	KDHBv_S05702_220870.t1	*Beta vulgaris*	similar to phosphoglucomutase (*Mesembryanthemum crystallinum*)	0.0	P93262	286/8/8	19	55.2/5.0	49.2/5.2	glucose metabolic process	GO:0006006
4308	KDHBv_S08411_274660.t1	*Beta vulgaris*	similar to triosephosphate isomerase (*Oryza coarctata*)	4.00E-158	B0LT90	202/4/4	21	27.4/5.4	32.8/6.1	glycolytic process	GO:0006096
4309	KDHBv_S08411_274660.t1	*Beta vulgaris*	similar to triosephosphate isomerase (*Oryza coarctata*)	4.00E-158	B0LT90	177/3/3	15	27.4/5.4	30.0/6.0	glycolytic process	GO:0006096
4402	CV301300	*Beta vulgaris*	similar to triosephosphate isomerase (*Capsicum annuum*)	8.00E-144	K4FXE7	471/10/7	36	27.0/7.8	33.3/5.9	glycolytic process	GO:0006096
3402	KDHBv_S01650_75980.t1	*Beta vulgaris*	similar to triosephosphate isomerase (*Spinacia oleracea*)	0.0	P48496	536/15/13	55	36.6/6.9	33.1/5.7	glycolytic process	GO:0006096
3503	KDHBv_S00669_241900.t2	*Beta vulgaris*	similar to fructose-bisphosphate aldolase (*Catharanthus roseus*)	0.0	F1AHC9	144/5/5	13	42.7/6.4	45.5/5.6	glycolytic process	GO:0006096
3506	KDHBv_S00669_241900.t2	*Beta vulgaris*	similar to fructose-bisphosphate aldolase (*Catharanthus roseus*)	0.0	F1AHC9	498/13/9	32	42.7/6.4	46.0/5.7	glycolytic process	GO:0006096
3507	Bv9_222620_wffm.t2	*Beta vulgaris*	similar to fructose-bisphosphate aldolase (*Catharanthus roseus*)	0.0	F1AHC9	282/5/5	23	33.3/5.9	46.7/5.5	glycolytic process	GO:0006096
3509	KDHBv_S00669_241900.t2	*Beta vulgaris*	similar to fructose-bisphosphate aldolase (*Catharanthus roseus*)	0.0	F1AHC9	253/9/9	30	427/6.4	44.5/5.8	glycolytic process	GO:0006096
4501	KDHBv_S00669_241900.t2	*Beta vulgaris*	similar to fructose-bisphosphate aldolase (*Catharanthus roseus*)	0.0	F1AHC9	413/11/10	41	42.7/6.4	46.3/5.9	glycolytic process	GO:0006096
4603	BI096185	*Beta vulgaris*	similar to fructose-bisphosphate aldolase (*Mesembryanthemum crystal*	7.00E-142	O04975	540/11/7	50	24.4/5.4	47.4/6.1	glycolytic process	GO:0006096
4604	BI096185	*Beta vulgaris*	similar to fructose-bisphosphate aldolase (*Mesembryanthemum crystal*	7.00E-142	O04975	538/10/8	44	24.4/5.4	48.8/5.9	glycolytic process	GO:0006096
5605	BI096185	*Beta vulgaris*	similar to fructose-bisphosphate aldolase (*Mesembryanthemum crystal*	7.00E-142	O04975	244/3/3	19	24.4/5.4	47.6/6.3	glycolytic process	GO:0006096
5607	BI096185	*Beta vulgaris*	similar to fructose-bisphosphate aldolase (*Mesembryanthemum crystal*	7.00E-142	O04975	547/10/7	51	24.4/5.4	48.5/6.3	glycolytic process	GO:0006096
5601	BI096185	*Beta vulgaris*	similar to fructose-bisphosphate aldolase (*Mesembryanthemum crystal*	7.00E-142	O04975	516/12/7	43	24.4/5.4	47.8/6.2	glycolytic process	GO:0006096
5503	KDHBv_S04922_201240.t1	*Beta vulgaris*	similar to fructose bisphosphate aldolase (*Salicornia herbacea*)	0.0	Q6RSN7	88/2/2	10	38.8/6.4	41.1/6.3	glycolytic process	GO:0006096
7605	gi|125662890	*Beta vulgaris*	glyceraldehyde-3-phosphate dehydogenase		A3FMH0	360/9/9	29	36.8/6.8	47.8/6.9	glucose metabolic process	GO:0006006
8603	gi|125662890	*Beta vulgaris*	glyceraldehyde-3-phosphate dehydogenase		A3FMH0	770/17/14	62	36.8/6.8	46.9/7.5	glucose metabolic process	GO:0006006
8601	gi|125662890	*Beta vulgaris*	glyceraldehyde-3-phosphate dehydogenase		A3FMH0	420/8/8	32	36.8/6.8	47.7/7.2	glucose metabolic process	GO:0006006
Pentose-phosphate shunt											
2409	Bv4_083150_jofx.t1	*Beta vulgaris*	similar to ribose-5-phosphate isomerase (*Spinacia oleracea*)	9.00E-156	Q8RU73	307/10/8	36	31.3/6.5	35.0/5.1	pentose-phosphate shunt, non-oxidative branch	GO:0009052
2404	Bv4_083150_jofx.t1	*Beta vulgaris*	similar to ribose-5-phosphate isomerase (*Spinacia oleracea*)	9.00E-156	Q8RU73	291/6/6	32	31.3/6.5	34.5/5.3	pentose-phosphate shunt, non-oxidative branch	GO:0009052
Calvin cycle/C fixation											
4305	Bv5_112800_cnci.t1	*Beta vulgaris*	similar to ribulose-phosphate 3-epimerase (*Spinacia oleracea*)	0.0	Q43157	217/6/6	34	30.6/7.7	31.9/6.2	Calvin cycle; pentose-phosphate shunt, oxidative branch	GO:0019253/ GO:0009052
4902	BQ587653	*Beta vulgaris*	similar to transketolase (*Spinacia oleracea*)	1.00E-98	O20250	189/4/4	24	22.7/7.2	103.8/6.0	Calvin cycle; pentose-phosphate shunt, oxidative branch	GO:0019253/ GO:0009052
5608	BQ587653	*Beta vulgaris*	similar to transketolase (*Spinacia oleracea*)	1.00E-98	O20250	230/5/5	28	22.7/7.2	57.8/6.2	Calvin cycle; pentose-phosphate shunt, oxidative branch	GO:0019253/ GO:0009052
5602	JK166473	*Arachis hypogaea*	similar to transketolase (*Platanus acerifolia*)	2.00E-99	Q14K68	88/2/2	20	18.9/6.3	62.3/6.2	Calvin cycle; pentose-phosphate shunt, oxidative branch	GO:0019253/ GO:0009052
1306	KDHBv_S02452_120120.t1	*Beta vulgaris*	similar to phosphoglycerate kinase (*Spinacia oleracea*)	0.0	P29409	236/7/7	21	50.9/6.7	31.9/4.9	Calvin cycle, glycolytic process	GO:0019253/ GO:0006096
1604	BQ588371	*Beta vulgaris*	sedoheptulose-1,7-bisphosphatase	2.00E-120	O20252	364/7/7	43	22.5/5.8	49.4/4.9	Calvin cycle	GO:0019253
3607	Bv5_103170_rsxd.t1	*Beta vulgaris*	similar to phosphoribulokinase (*Spinacia oleracea*)	0.0	P09559	352/10/9	28	45.5/5.9	50.0/5.7	Calvin cycle	GO:0019253
7208	BQ586293	*Beta vulgaris*	similar to ribulose-1,5-bisphosphate carboxylase/oxygenase (*Mesembr*	1.00E-84	Q08184	84/3/3	12	25.3/8.9	20.5/7.0	carbon fixation	GO:0015977

3807	Bv3_055610_usdd.t1	*Beta vulgaris*	similar to ribulose-1,5-bisphosphate carboxylase/oxygenase (*Cucumis*	0.0	*XP_004144069*	1084/27/21	47	64.7/6.0	78.0/5.6	carbon fixation	GO:0015977
7402	DN911533	*Beta vulgaris*	ribulose-1,5-bisphosphate carboxylase/oxygenase	3.00E-82	P16032	97/3/3	11	22.2/9.5	34.3/7.0	carbon fixation	GO:0015977
7705	tr|Q6JXV6|Q6JXV6_BETVU	*Beta vulgaris*	ribulose-1,5-bisphosphate carboxylase/oxygenase	0.0	A0A023ZPS4	591/26/17	44	50.0/6.1	66.0/6.9	carbon fixation	GO:0015977
1605	tr|Q4PLI7|Q4PLI7_BETVU	*Beta vulgaris*	ribulose-1,5-bisphosphate carboxylase/oxygenase	4.00E-172	A0A023ZPS4	103/4/3	15	26.7/6.7	60.2/4.9	carbon fixation	GO:0015977
5706	gi|34576609	*Beta vulgaris*	ribulose-1,5-bisphosphate carboxylase/oxygenase		Q6JXV6	833/19/14	38	50.0/6.1	66.3/6.4	carbon fixation	GO:0015977
Photosyntesis-light harvesting											
3203	DV501827	*Beta vulgaris*	similar to 23 kDa OEC protein (*Salicornia veneta*)	1.00E-79	B0L802	385/7/7	36	22.6/8.8	28.5/5.7	photosynthesis	GO:0015979
4307	DV501827	*Beta vulgaris*	similar to 23 kDa OEC protein (*Salicornia veneta*)	2.00E-79	B0L802	216/5/5	26	22.6/8.8	28.7/5.9	photosynthesis	GO:0015979
3302	DV501827	*Beta vulgaris*	similar to 23 kDa OEC protein (*Salicornia veneta*)	1.00E-79	B0L802	95/3/3	15	22.6/8.8	30.5/5.6	photosynthesis	GO:0015979
Tricarboxylic acid pathway											
4503	gi|11133601	*Beta vulgaris*	malate dehydrogenase, cytoplasmic		Q9SML8	538/12/9	34	35.8/5.9	45.9/6.1	cellular carbohydrate metabolic process	GO:0044262
4504	gi|11133601	*Beta vulgaris*	malate dehydrogenase, cytoplasmic		Q9SML8	792/17/12	47	35.8/5.9	46.6/6.2	cellular carbohydrate metabolic process	GO:0044262
5606	gi|11133601	*Beta vulgaris*	malate dehydrogenase, cytoplasmic		Q9SML8	887/25/14	50	35.8/5.9	47.4/6.5	cellular carbohydrate metabolic process	GO:0044262
7504	gi|11133601	*Beta vulgaris*	malate dehydrogenase, cytoplasmic		Q9SML8	240/4/4	15	35.8/5.9	45.0/6.8	cellular carbohydrate metabolic process	GO:0044262
7601	gi|11133601	*Beta vulgaris*	malate dehydrogenase, cytoplasmic		Q9SML8	201/3/3	12	35.8/5.9	47.2/6.8	cellular carbohydrate metabolic process	GO:0044262
one carbon metabolism											
7304	AW063033	*Beta vulgaris*	similar to carbonic anhydrase (*Spinacia oleracea*)	5.00E-132	P16016	123/4/3	15	32.7/8.3	31.9/7.0	one-carbon metabolic process	GO:0006730
7302	Bv8_198910_rkme.t1	*Beta vulgaris*	similar to carbonic anhydrase (*Spinacia oleracea*)	0.0	P16016	246/9/7	32	35.8/6.9	32.3/6.9	one-carbon metabolic process	GO:0006730
5412	YTiBv_S01345_54430.t2	*Beta vulgaris*	similar to carbonic anhydrase (*Spinacia oleracea*)	0.0	P16016	394/16/9	38	36.0/6.9	33.5/6.4	one-carbon metabolic process	GO:0006730
8302	Bv9_212920_hdnw.t1	*Beta vulgaris*	similar to carbonic anhydrase (*Spinacia oleracea*)	0.0	P16016	128/4/4	15	36.0/6.9	32.0/7.4	one-carbon metabolic process	GO:0006730
4405	BQ590397	*Beta vulgaris*	similar to putative carbonic anhydrase (*Populus trichocarpa*)	9.00E-61	U5GK55	246/6/6	38	22.8/9.0	37.3/6.1	response to oxidative stress	GO:0006979
carbon others											
5508	AW697745	*Beta vulgaris*	similar to oxaloacetase (*Dianthus caryophyllus*)	2.00E-105	Q05957	99/3/3	25	25.4/8.7	44.6/6.4	metabolic process	GO:0008152
**Polysacharide metabolism (16 spots, 10 Uniprot entries)**											
506	BQ587102	*Beta vulgaris*	similar to 3-glucanase family protein (*Populus trichocarpa*)	9.00E-55	B9GI31	129/5/2	16	24.2/8.3	43.4/4.2	carbohydrate metabolic process	GO:0005975
504	KDHBv_S01757_83500.t1	*Beta vulgaris*	similar to 3-glucanase family protein (*Populus trichocarpa*)	2.00E-127	B9GI31	701/22/10	44	37.2/4.5	46.0/4.2	carbohydrate metabolic process	GO:0005975
1504	gi|1168935	*Beta vulgaris*	acidic endochitinase SP2		P42820	637/13/10	50	31.2/5.0	42.4/4.8	polysaccharide catabolic process	GO:0000272
1506	gi|1168935	*Beta vulgaris*	acidic endochitinase SP2		P42820	659/17/11	52	31.2/5.0	40.9/4.8	polysaccharide catabolic process	GO:0000272
304	gi|544000	*Beta vulgaris*	acidic endochitinase SE2		P36910	143/3/3	9	31.1/4.3	31.8/4.0	polysaccharide catabolic process	GO:0000272
7404	Bv5_112700_mohc.t1	*Beta vulgaris*	similar to chitinase (*Phytolacca americana*)	7.00E-154	Q8LST3	382/13/7	24	33.1/8.1	34.7/7.1	carbohydrate metabolic process	GO:0005975
8401	Bv5_112700_mohc.t1	*Beta vulgaris*	similar to chitinase (*Phytolacca americana*)	7.00E-154	Q8LST3	715/19/10	40	33.1/8.1	34.5/7.6	carbohydrate metabolic process	GO:0005975
7406	BQ585684	*Beta vulgaris*	similar to chitinase (*Phytolacca americana*)	1.00E-89	Q8LST3	153/3/3	24	23.6/6.6	35.6/6.7	carbohydrate metabolic process	GO:0005975
9403	KDHBv_S14175_58500.t1	*Beta vulgaris*	similar to chitinase (*Phytolacca americana*)	2E-154	Q8LST3	262/8/7	28	33.0/8.6	34.5/8.2	carbohydrate metabolic process	GO:0005975
3809	FG345333	*Beta vulgaris*	similar to uncharacterized protein (*Citrus clementina*)	4.00E-143	V4SY44	419/9/7	41	28.2/4.9	96.7/5.7	carbohydrate metabolic process	GO:0005975
5507	FQ326228	*Casuarina glauca*	similar to unknown protein (*Populus trichocarpa*)	2.00E-35	A9PG55	88/2/1	9	15.2/9.5	41.5/6.2	mannose metabolic process	GO:0006013
3805	KDHBv_S08054_268480.t1	*Beta vulgaris*	similar to beta-xylosidase/alpha-L-arabinofuranosidase (*Prunus mume*	0.0	*XP_008218886*	169/7/7	13	85.4/6.8	97.2/5.6	xylan catabolic process	GO:0045493
4901	KDHBv_S08054_268480.t1	*Beta vulgaris*	similar to beta-xylosidase/alpha-L-arabinofuranosidase (*Prunus mume*	0.0	*XP_008218886*	591/21/19	33	85.4/6.8	103.8/5.8	xylan catabolic process	GO:0045493

6504	KDHBv_S05428_214020.t1	*Beta vulgaris*	similar to UDP-glucuronic acid decarboxylase 1 (*Morus notabilis*)	0.0	W9R277	182/6/6	21	38.6/6.7	45.1/6.6	UDP-D-xylose biosynthetic process	GO:0033320
1601	gi|18072857	*Beta vulgaris*	beta-fructofuranosidase		Q8VXS6	182/6/4	15	32.0/4.9	60.9/4.8	carbohydrate metabolic process	GO:0005975
1804	Bv3_056730_iafj.t1	*Beta vulgaris*	similar to beta-fructofuranosidase (*red goosefoot*)	0.0	S49256	236/7/6	16	51.3/4.7	94.5/5.0	carbohydrate metabolic process	GO:0005975
**Protein metabolism (24 spots, 21 Uniprot entries)**											
Proteolysis											
1304	KDHBv_S01086_20800.t1	*Beta vulgaris*	similar to proteasome subunit alpha type-5 (*Glycine max*)	8.00E-161	Q9M4T8	146/7/6	35	26.2/4.8	32.2/4.6	ubiquitin-dependent protein catabolic process	GO:0006511
3303	KDHBv_S00322_151230.t1	*Beta vulgaris*	similar to predicted proteasome subunit alpha type (*Vitis vinifera*)	2.00E-157	D7T9I6	285/9/9	48	25.6/5.4	32.9/5.6	ubiquitin-dependent protein catabolic process	GO:0006511
5414	KDHBv_S11207_25220.t1	*Beta vulgaris*	similar to proteasome subunit alpha type-6-like isoform X1 (*Cicer ari*	6.00E-158	*XP_004488480*	433/14/10	51	27.3/6.0	33.9/6.3	ubiquitin-dependent protein catabolic process	GO:0006511
7405	gi|392938146	*Beta vulgaris*	Proteasome subunit alpha type		I6U5E4	464/12/9	44	27.3/6.7	33.3/6.8	ubiquitin-dependent protein catabolic process	GO:0006511
9303	Bv6_127070_exia.t1	*Beta vulgaris*	similar to proteasome subunit alpha type-7 (*Malus domestica*)	9.00E-142	*XP_008393990*	140/5/5	24	27.0/6.9	31.2/8.0	ubiquitin-dependent protein catabolic process	GO:0006511
2302	KDHBv_S00423_183180.t1	*Beta vulgaris*	similar to proteasome subunit beta type-6 (*Brassica rapa*)	1.00E-130	M4D453	120/3/3	16	25.4/4.9	30.5/5.2	ubiquitin-dependent protein catabolic process	GO:0006511
1402	BQ488414	*Beta vulgaris*	similar to cysteine proteinase RD19a/like (Glycine max)	7.00E-65	I1LJ95	145/4/3	18.0	21.6/5.7	38.3/4.8	proteolysis	GO:0006508
405	YTiBv_S08134_271690.t1	*Beta vulgaris*	similar to cysteine protease (*Actinidia deliciosa*)	0.0	A5HIJ1	177/5/4	17	39.9/5.7	34.5/4.3	proteolysis	GO:0006508
8605	DV501732	*Beta vulgaris*	similar to aspartic protease (*Citrus sinensis*)	3.00E-33	A0A067FW02	211/6/3	28	12.9/7.5	47.3/7.6	proteolysis	GO:0006508
3609	KDHBv_S01306_46650.t1	*Beta vulgaris*	similar to serine carboxypeptidase (*Morus notabilis*)	0.0	W9SXH8	139/3/3	10	44.7/5.1	59.1/5.7	proteolysis	GO:0006508
7207	KDHBv_S03070_144690.t1	*Beta vulgaris*	similar to serine carboxypeptidase (*Ricinus communis*)	0.0	B9SMP4	162/4/3	9	56.6/5.5	23.3/7.0	proteolysis	GO:0006508
1401	BQ594321	*Beta vulgaris*	similar to serine carboxypeptidase-like 20-like (*Prunus mume*)	1.00E-72	*XP_008235895*	288/6/5	26	21.8/5.7	33.3/4.9	proteolysis	GO:0006508
1501	BQ594321	*Beta vulgaris*	similar to serine carboxypeptidase-like 20-like (*Prunus mume*)	1.00E-72	*XP_008235895*	207/4/4	26	21.8/5.7	41.1/4.5	proteolysis	GO:0006508
4801	DV501590	*Beta vulgaris*	similar to unknown protein (*Vitis vinifera*)	8.00E-82	E0CQB3	321/7/5	34	21.1/7.6	98.5/6.1	serine-type endopeptidase activity	GO:0004252
4906	DV501590	*Beta vulgaris*	similar to unknown protein (*Vitis vinifera*)	8.00E-82	E0CQB3	272/5/4	27	21.1/7.6	104.6/6.1	serine-type endopeptidase activity	GO:0004252
8901	KDHBv_S05612_218410.t1	*Beta vulgaris*	similar to predicted subtilisin-like protease-like (*Citrus sinensis*)	0.0	*XP_006466502*	262/7/6	11	77.8/6.2	105.6/7.1	serine-type peptidase activity	GO:0008236
Protein folding											
3301	Bv8_194790_iuxp.t1	*Beta vulgaris*	similar to chaperonin 20 (*Theobroma cacao*)	3.00E-133	A0A061GL19	395/11/7	48	26.9/8.5	31.9/5.6	protein folding	GO:0006457
2204	FG345335	*Beta vulgaris*	similar to peptidyl-prolyl cis-trans isomerase (*Ricinus communis*)	4.00E-67	B9RN18	281/5/4	21	24.1/8.5	23.6/5.4	protein folding	GO:0006457
2203	KDHBv_S01128_26190.t1	*Beta vulgaris*	similar to peptidyl-prolyl cis-trans isomerase (*Ricinus communis*)	0.0	B9RN18	216/4/4	21	28.3/7.6	23.5/5.2	protein folding	GO:0006457
1602	KDHBv_S12447_40980.t1	*Beta vulgaris*	similar to peptidyl-prolyl cis-trans isomerase (*Spinacia oleracea*)	0.0	O49939	452/17/13	46	42.4/4.72	51.1/4.9	protein folding	GO:0006457
1802	KDHBv_S00813_270460.t1	*Beta vulgaris*	similar to rubisco subunit binding-protein alpha subunit (*Populus trich*	0.0	B9MZ75	1331/34/22	62	50.7/4.8	83.1/4.9	protein folding	GO:0006457
2902	KDHBv_S00726_253620.t1	*Beta vulgaris*	similar to heat shock 70 protein (*Spinacia oleracea*)	0.0	O22664	375/12/11	21	71.7/5.1	101.9/5.3	protein folding	GO:0006457
2904	KDHBv_S00726_253620.t1	*Beta vulgaris*	similar to predicted heat shock cognate 70 kDa protein 2-like (*Cicer a*	0.0	*XP_004505872*	199/4/4	9	71.6/5.2	102.3/5.5	protein folding	GO:0006457
Translation											
5306	KDHBv_S02429_119490.t1	*Beta vulgaris*	similar to elongation factor Tu (*Citrus sinensis*)	0.0	A0A067FTF8	249/5/4	11	53.3/6.5	30.8/6.3	translation	GO:0006412
**Amino acid metabolism (10 spots, 7 Uniprot entries)**											
8710	Bv3_067820_eeis.t1	*Beta vulgaris*	similar to serine hydroxymethyltransferase (*Theobroma cacao*)	0.0	*XP_007034218*	700/21/16	47	52.5/7.1	67.9/7.6	L-serine metabolic process	GO:0006563
8705	Bv3_067820_eeis.t1	*Beta vulgaris*	similar to serine hydroxymethyltransferase (*Theobroma cacao*)	0.0	*XP_007034218*	388/11/10	33	52.5/7.1	68.2/7.4	L-serine metabolic process	GO:0006563
8711	CV301395	*Beta vulgaris*	similar to serine hydroxymethyltransferase (*Theobroma cacao*)	2.00E-171	*XP_007034219*	119/3/3	15	29.1/8.8	70.0/7.3	L-serine metabolic process	GO:0006563
7706	Bv6_152120_wtfn.t1	*Beta vulgaris*	similar to serine transhydroxymethyltransferase (*Solanum tuberosum*)	0.0	P50433	704/24/21	57	57.3/7.2	67.8/7.1	L-serine metabolic process	GO:0006563
9602	FG343537	*Beta vulgaris*	similar to aminomethyltransferase (*Mesembryanthemum crystallinum*)	3.00E-118	P93256	107/2/2	12	21.4/6.5	49.0/8.2	glycine catabolic process	GO:0006546
2609	gi|12963877	*Beta vulgaris*	glutamine synthetase		Q9AWA8	439/9/7	22	47.9/5.7	56.3/5.4	glutamine biosynthetic process	GO:0006542
3602	gi|12963877	*Beta vulgaris*	glutamine synthetase		Q9AWA8	223/4/4	14	47.9/5.7	53.9/5.5	glutamine biosynthetic process	GO:0006542
2610	gi|13173419	*Beta vulgaris*	glutamine synthetase		Q9AXD1	201/5/5	16	39.2/5.3	51.3/5.5	glutamine biosynthetic process	GO:0006542

6602	KDHBv_S02422_118980.t1	*Beta vulgaris*	similar to aspartate aminotransferase (*Populus trichocarpa*)	0.0	B9HAW0	505/14/13	33	50.7/8.1	52.6/6.6	cellular amino acid metabolic process	GO:0006520
7602	KDHBv_S02422_118980.t1	*Beta vulgaris*	similar to aspartate aminotransferase (*Populus trichocarpa*)	0.0	B9HAW0	394/15/14	34	50.7/8.1	51.7/6.9	cellular amino acid metabolic process	GO:0006520
**Stress related (28 spots, 23 Uniprot entries)**											
Defense											
302	BQ488612	*Beta vulgaris*	similar to osmotin-like protein (*Atriplex nummularia*)	4.00E-71	Q38745	234/8/3	24	21.8/4.5	29.4/3.9	defense response	GO:0006952
303	BQ488612	*Beta vulgaris*	similar to osmotin-like protein (*Atriplex nummularia*)	4.00E-71	Q38745	211/6/4	35	21.8/4.5	30.0/4.0	defense response	GO:0006952
8303	CK136649	*Beta vulgaris*	similar to osmotin-like protein (*Atriplex nummularia*)	2.00E-79	Q38745	180/3/3	11	32.2/8.6	28.9/7.6	defense response	GO:0006952
9204	BQ585420	*Beta vulgaris*	similar to thaumatin-like protein (*Mirabilis jalapa*)	2.00E-74	Q6PP01	80/2/2	21	18.5/8.9	27.7/7.9	defense response	GO:0006952
5307	Bv5_095620_hefa.t1	*Beta vulgaris*	similar to thaumatin like protein (*Nepenthes alata*)	3.00E-116	A9ZMG1	98/2/2	11	25.0/8.1	28.8/6.4	defense response	GO:0006952
8203	KDHBv_S02192_109120.t1	*Beta vulgaris*	similar to thaumatin like protein (*Nepenthes alata*)	2.00E-118	A9ZMG0	248/6/5	34	25.0/7.8	27.3/7.4	defense response	GO:0006952
1404	BQ584258	*Beta vulgaris*	similar to thaumatin/like protein 1 (*Fragaria vesca*)	2.00E-44	*XP_004297839*	104/2/2	17	17.4/5.2	37.3/4.6	defense response	GO:0006952
2613	Bv1_013190_noyh.t1	*Beta vulgaris*	similar to abscisic acid stress ripening-related protein (*Suaeda liaotung*	3.00E-17	A0A059SPX5	104/2/2	15	26.1/5.1	61.6/5.1	response to stress	GO:0006950
2408	Bv1u_019150_prnw.t1	*Beta vulgaris*	similar to protein IN2-1 homolog B-like (*Vitis vinifera*)	4.00E-109	*XP_003632205*	222/9/9	59	26.8/5.0	33.6/5.1	glutathione metabolic process	GO:0006749
1201	BQ586089	*Beta vulgaris*	similar to uncharacterized protein *(Solanum tuberosum)*	3.00E-29	M0ZYA5	259/5/5	29	18.9/6.5	20.5/4.8	defense response	GO:0006952
Redox defense											
4406	Bv9_226730_qcfr.t1	*Beta vulgaris*	similar to ascorbate peroxidase (*Suaeda salsa*)	1.00E-139	Q94CF7	118/4/4	24	27.7/5.5	35.6/6.0	response to oxidative stress	GO:0006979
2614	Bv4_071810_dkmh.t1	*Beta vulgaris*	similar to peroxidase superfamily protein (*Theobroma cacao*)	2.00E-154	*XP_007014796*	233/7/6	23	36.2/6.0	48.6/5.3	response to oxidative stress	GO:0006979
8608	Bv1_013700_wnij.t1	*Beta vulgaris*	similar to peroxidase (*Spinacia oleracea*)	0.0	P93547	382/10/9	33	39.0/6.6	59.5/7.1	response to oxidative stress	GO:0006979
4602	BI073121	*Beta vulgaris*	similar to monodehydroascorbate reductase (*Mesembryanthemum crys*	2.00E-123	Q93YG1	400/7/6	37	23.9/6.7	58.3/6.1	response to oxidative stress	GO:0006979
2201	KDHBv_S08545_277370.t1	*Beta vulgaris*	similar to peroxiredoxin (*Tamarix hispida*)	3.00E-143	H6VND7	356/12/7	35	29.8/8.4	27.2/5.0	cell redox homeostasis	GO:0045454
2202	KDHBv_S08545_277370.t1	*Beta vulgaris*	similar to peroxiredoxin (*Tamarix hispida*)	3.00E-143	H6VND7	93/4/4	16	29.8/8.4	28.1/5.1	cell redox homeostasis	GO:0045454
2608	AW067625	*Beta vulgaris*	similar to peroxiredoxin (*Tamarix hispida*)	6.00E-43	H6VND7	154/3/3	12	36.1/9.4	56.0/5.3	cell redox homeostasis	GO:0045454
7209	FG343498	*Beta vulgaris*	similar to type II peroxiredoxin (*Tamarix hispida*)	1.00E-82	I0CC96	78/2/2	11	18.2/8.8	25.0/6.9	cell redox homeostasis	GO:0045454
3201	Bv9_210170_xpds.t1	*Beta vulgaris*	similar to Cu/Zn superoxide dismutase (*Salicornia europaea*)	7.00E-98	H9BQP8	203/5/4	27	23.2/5.7	22.2/5.5	superoxide metabolic process	GO:0006801
3504	BQ587104	*Beta vulgaris*	similar to predicted isoflavone reductase homolog (*Malus domestica*)	8.00E-106	*XP_008377292*	371/8/7	35	24.2/9.2	44.2/5.6	oxidation-reduction process	GO:0055114
Methylglyoxal detoxification											
2407	YMoBv_S03608_189140.t1	*Beta vulgaris*	similar to lactoylglutathione lyase (*Solanum tuberosum*)	0.0	M0ZHD0	174/8/8	29	38.7/6.9	38.0/5.4	methylglyoxal catabolic process to D-lactate	GO:0019243
3501	YTiBv_S03077_156120.t1	*Beta vulgaris*	similar to lactoylglutathione lyase (*Solanum tuberosum*)	0.0	M0ZHD0	271/8/7	26	38.7/6.9	38.4/5.6	methylglyoxal catabolic process to D-lactate	GO:0019243
2503	KDHBv_S04734_195510.t1	*Beta vulgaris*	similar to lactoylglutathione lyase (*Gossypium hirsutum)*	0.0	D2D330	622/27/10	38	32.6/5.2	45.6/5.3	methylglyoxal catabolic process to D-lactate	GO:0019243
2506	FG343264	*Beta vulgaris*	similar to lactoylglutathione lyase (*Arabidopsis thaliana*)	1.00E-120	Q8W593	231/6/5	34	21.3/5.4	39.6/5.4	methylglyoxal catabolic process to D-lactate	GO:0019248
2205	YMoBv_S05969_245200.t2	*Beta vulgaris*	similar to lactoylglutathione lyase isoform X2 (*Pyrus x bretschneideri*	4.00E-120	*GI:694332574*	260/11/9	49	27.2/7.8	28.3/5.4	methylglyoxal catabolic process to D-lactate	GO:0019244
2208	Bv9_209300_pjcz.t1	*Beta vulgaris*	similar to lactoylglutathione lyase isoform X2 (*Malus domestica*)	2.00E-120	XP_008385524	56/2/2	10	20.9/5.4	28.2/5.2	methylglyoxal catabolic process to D-lactate	GO:0019245
2505	KDHBv_S04734_195510.t1	*Beta vulgaris*	similar to lactoylglutathione lyase (*Nicotiana tomentosiformis*)	0.0	*GI:697188226*	314/9/8	33	32.6/5.2	45.1/5.4	methylglyoxal catabolic process to D-lactate	GO:0019246
3508	YMoBv_S03608_189140.t1	*Beta vulgaris*	similar to lactoylglutathione lyase (*Nicotiana tomentosiformis*)	0.0	*GI:697141977*	464/13/12	41	38.7/6.9	39.7/5.6	methylglyoxal catabolic process to D-lactate	GO:0019247
**Lipid metabolism (3 spots, 3 Uniprot entries)**											
3606	BQ489322	*Beta vulgaris*	similar to 3-hydroxybutyryl-CoA dehydratase (*Ricinus communis*)	1.00E-29	B9RPB0	116/2/2	24	10.5/7.9	53.5/5.6	enoyl-CoA hydratase activity	GO:0004300
8604	BQ587656	*Beta vulgaris*	similar to uncharacterized protein (*Populus trichocarpa*)	2.00E-73	U5FE87	266/4/3	21	24.5/8.8	50.4/7.2	lipid metabolic process	GO:0006629
1502	BQ586653	*Beta vulgaris*	similar to predicted uncharacterized protein (*Vitis vinifera*)	3.00E-89	D7TJU3	299/4/4	28	23.9/4.8	46.8/4.7	lipid metabolic process	GO:0006629
**Energy:oxisoreductases-electron transport (7 spots, 4 Uniprot entries)**											
6502	BQ587298	*Beta vulgaris*	similar to ferredoxin--NADP reductase (*Ricinus communis*)	7.00E-140	B9SB31	207/4/4	26	24.5/5.6	43.2/6.7	oxidation-reduction process	GO:0055114
3611	YMoBv_S01806_99510.t1	*Beta vulgaris*	similar to predicted alcohol dehydrogenase (*Ricinus communis*)	3.00E-166	B9SHB0	663/19/16	61	34.5/5.3	46.9/5.6	oxidation-reduction process	GO:0055114

3601	KDHBv_S08270_272740.t1	*Beta vulgaris*	similar to predicted alcohol dehydrogenase (*Ricinus communis*)	1.00E-168	B9SHB0	495/18/17	53	41.0/6.8	48.1/5.5	oxidation-reduction process	GO:0055114
3605	KDHBv_S08270_272740.t1	*Beta vulgaris*	similar to predicted alcohol dehydrogenase (*Ricinus communis*)	1.00E-168	B9SHB0	750/25/20	55	41.0/6.8	48.0/5.6	oxidation-reduction process	GO:0055114
4303	KDHBv_S15268_67460.t1	*Beta vulgaris*	similar to flavoprotein WrbA-like (*Fragaria vesca*)	1.00E-114	*XP_004294313*	120/4/4	24	20.4/5.7	29.7/6.1	oxidation-reduction process	GO:0055114
**Other (5 spots, 5 Uniprot entries)**											
5604	gi|340629183	*s tremula x Popul*	cytosolic ATP sulfurylase		G9B7N0	341/8/6	20	36.2/6.4	61.0/6.4	sulfate assimilation	GO:0000103
6110	KDHBv_S03162_148190.t1	*Beta vulgaris*	similar to nucleoside diphosphate kinase 2 (*Spinacia oleracea*)	1.00E-126	Q01402	120/4/6	10	26.2/8.5	19.8/6.6	nucleotide metabolic process	GO:0009117
2501	Bv2_046480_auis.t1	*Beta vulgaris*	similar to thiamine thiazole synthase (*Prunus mume*)	0.0	*XP_008244366*	100/5/4	17	37.7/5.0	39.0/5.1	thiamine biosynthetic process	GO:0009228
7503	BQ488773	*Beta vulgaris*	similar to uncharacterized germin protein (*Medicago truncatula*)	2.00E-81	I3SGS4	89/2/2	24	18.0/6.4	43.0/6.9	nutrient reservoir activity	GO:0045735
2406	KDHBv_S06018_227140.t1	*Beta vulgaris*	similar to soluble inorganic pyrophosphatase 1 (*Theobroma cacao*)	5.00E-169	A0A061E4X1	247/9/7	28	33.5/7.7	37.1/5.3	pyrophosphatase activity	GO:0016462
2303	KDHBv_S09907_301830.t1	*Beta vulgaris*	similar to acylpyruvase FAHD1 (*Cicer arietinum*)	8.00E-103	*XP_004508199*	436/21/8	49	23.5/5.3	30.9/5.4	hydrolase activity	GO:0016787
2304	Bv7u_181350_jrmq.t1	*Beta vulgaris*	similar to acylpyruvase FAHD1 (*Cicer arietinum*)	4.00E-102	*XP_004508199*	83/4/4	22	23.5/5.3	31.3/5.3	hydrolase activity	GO:0016787
**Unknown function (12 spots, 9 Uniprot entries)**											
2103	Bv9_223610_jrfd.t1	*Beta vulgaris*	similar to putative protein (*Hordeum vulgare*)	2.00E-39	F2EID0	155/3/3	25	16.5/6.3	17.9/5.4	nucleotide binding	GO:0000166
7303	UMSBv_S06580_303800.t1	*Beta vulgaris*	similar to uncharacterized protein (*Vitis vinifera*)	9.00E-98	D7SXW6	301/11/8	50	25.3/6.5	30.5/6.9		
8205	YMoBv_S00976_306700.t1	*Beta vulgaris*	similar to uncharacterized protein (*Vitis vinifera*)	3.00E-98	D7SXW6	165/4/4	21	25.1/6.5	28.0/7.7		
9304	YMoBv_S00976_306700.t1	*Beta vulgaris*	similar to uncharacterized protein (*Vitis vinifera*)	3.00E-98	D7SXW6	132/4/4	23	25.1/6.5	30.8/7.7		
6307	YMoBv_S00976_306700.t1	*Beta vulgaris*	similar to uncharacterized protein (*Vitis vinifera*)	3.00E-98	D7SXW6	290/8/8	52	25.1/6.5	29.5/6.6		
3610	UMSBv_S26183_171390.t1	*Beta vulgaris*	similar to uncharacterized protein (*Jatropha curcas*)	4.00E-57	A0A067KSH6	422/14/10	42	40.2/5.5	62.9/5.8		
6402	KDHBv_S02053_102420.t1	*Beta vulgaris*	similar to uncharacterized protein (*Jatropha curcas*)	2.00E-150	A0A067KHD5	307/8/7	36	275/5.8	35.0/6.6		
1104	CK136200	*Beta vulgaris*	similar to jasmonate-induced protein homolog (*Atriplex canescens*)	3.00E-56	P42764	145/3/2	10	29.6/8.5	20.1/5.0		
6601	gi|312273913	*Silene vulgaris*	CSP41A protein		E5KGE2	314/9/7	29	33.9/5.4	47.3/6.6	cellular metabolic process	GO:0044237
3206	GD575989	*haseolus coccineus*				65/2/1	34	3.7/10.4	22.8/5.6		
1202	KDHBv_S04042_178280.t1	*Beta vulgaris*				195/5/5	37	21.4/4.8	21.1/4.6		
7206	BQ586065	*Beta vulgaris*	hypothetical protein CICLE_v10029208mg (*Citrus clementina*)	7.00E-72	V4SBG5	125/2/2	14	19.9/6.2	28.3/7.0		
**No identified (45 spots)**											
202			no id						26.1/3.6		
204			no id						27.5/4.2		
205			no id						23.1/3.8		
502			no id						41.0/3.4		
803			no id						72.3/4.3		
1205			no id						27.3/4.9		
1303			no id						32.0/4.5		
1406			no id						34.4/4.8		
1603			no id						53.9/4.9		
1901			no id						103.0/4.8		
2207			no id						21.9/5.3		
2301			no id						32.8/5.1		
2401			no id						37.6/5.1		
2402			no id						37.5/5.2		
2405			no id						36.1/5.3		
2502			no id						38.7/5.2		
2602			no id						57.2/5.1		
2603			no id						51.8/5.1		
2801			no id						93.1/5.1		
3204			no id						26.7/5.6		
3205			no id						23.3/5.5		
4204			no id						20.4/6.1		
4408			no id						35.6/6.2		
4601			no id						58.6/6.0		
5202			no id						20.6/6.3		
5203			no id						28.0/6.5		
6202			no id						28.4/6.6		
6204			no id						23.6/6.7		
6306			no id						32.7/6.7		
6503			no id						41.2/6.6		
6703			no id						67.2/6.6		
7202			no id						27.4/6.9		

7204	no id	26.7/6.9
7401	no id	33.4/7.0
7403	no id	35.9/6.9
7604	no id	48.4/7.1
8206	no id	28.0/7.2
8304	no id	30.9/7.5
8403	no id	38.3/7.7
8404	no id	36.1/7.4
8406	no id	33.8/7.4
8502	no id	40.4/7.1
8503	no id	42.1/7.4
9501	no id	41.2/7.9
9605	no id	49.2/7.9
